# FullSynesth: Syntenic Reconciliation of a Set of Consistent Gene Trees

**DOI:** 10.1007/s00224-025-10259-2

**Published:** 2026-02-10

**Authors:** Mathieu Gascon, Mattéo Delabre, Nadia El-Mabrouk

**Affiliations:** Département d’informatique et de recherche opérationnelle, Université de Montréal, Québec, Canada

**Keywords:** Phylogenetics, Gene, Supertree, Reconciliation, Synteny, Duplication, Transfer, Loss

## Abstract

We present *FullSynesth*, a tree reconciliation algorithm predicting the evolution of a set of homologous genomic regions or *syntenies*, inside a species tree. The considered evolutionary model involves *segmental events* (i.e. acting on multiple genes) including duplications (D), losses (L), synteny fissions and transfers possibly going through unsampled or extinct species. Formally, given a set of syntenies in a set of genomes and a set $\mathcal {G}$ of consistent gene trees for the gene families composing the syntenies, the problem is to infer a most parsimonious evolutionary history explaining the observed gene trees and syntenies given a species tree. The problem is known to be NP-hard for the DL distance. FullSynesth is based on *Synesth* explicating the evolution of a set of syntenies given a single *synteny tree*, which can be obtained from $\mathcal {G}$ by selecting a given supertree. Rather than trying each supertree in turn, FullSynesth is based on a two-in-one approach simultaneously building and reconciling a *synteny supertree*. This algorithm runs in polynomial time for a fixed number of gene trees. We show on simulated datasets that FullSynesth significantly improves the running time of Synesth applied to each possible supertree. An implementation of the algorithm is available at: https://github.com/UdeM-LBIT/FullSynesth.

## Introduction

A *gene/species trees reconciliation* is an embedding of a gene tree into a species tree explaining the difference between the two trees through a sequence of events shaping the gene family inside the species tree. Reconciliation has been widely studied [1], first focusing on duplications (D) and losses (L), then extending to horizontal gene transfers (HGT) and other events [2, 3]. Finding a minimum Duplication-Transfer-Loss (DTL) scenario is NP-hard if time-inconsistencies in terms of cyclic inferences are forbidden, but it becomes polynomial if acyclicity requirements are relaxed [2, 3]. Various software tools have been developed (e.g. Ranger-DTL [4], Eucalypt [5], ecceTERA [6]). However, a major drawback of classical reconciliation is that gene families are considered separately from one another, which is not appropriate for genes organized in *syntenies*, i.e. colocalized genes evolving together through segmental events. This is the case of several operons such as the one containing the Cas genes of the CRISPR-Cas systems allowing prokaryotes to defend against invading viruses and plasmids [7]. Various methods have been developed to infer the evolution of adjacencies [8], group individual events into segmental ones [9], or minimize duplication episodes [10, 11], but none of them are intended to explicitly look for evolutionary scenarios with segmental events.

We presented the first algorithm generalizing reconciliation to segmental events and synteny trees (i.e. with leaves representing syntenies rather than single genes) for the Duplication-Loss (DL) distance in [12], for the Duplication-Transfer-Loss (DTL) distance in [13], and more recently for a more flexible evolutionary model also involving cuts (fissions) and the possibility of transient events going through unsampled or extinct species [14]. This latter algorithm is called *Synesth* (for *SYNteny Evolution in SegmenTal Histories*).

For any tree reconciliation method, obtaining accurate input trees is crucial. This is particularly challenging in the case of a reconciliation model requiring a synteny tree as input, as phylogenetic studies usually lead to gene trees. If the individual gene trees are consistent, then a supertree (a tree displaying them all) can be obtained [15–17]. We call a *synteny supertree* such a supertree leaf-labeled with the corresponding syntenies. However, the number of such synteny supertrees can be exponential in the number of syntenies.

In this paper, given a species tree *S*, a set $\mathcal {F}$ of gene families, a set ${\mathcal {X}}$ of syntenies which are subsets of $\mathcal {F}$ (i.e. syntenies are considered to be unordered), and a set of consistent gene trees $\mathcal {G}= \{G_1, G_2, \dots , G_k\}$ for each gene family of $\mathcal {F}$, we seek a synteny supertree for $\mathcal {G}$ leading to a most parsimonious reconciliation with *S* explaining the evolution of ${\mathcal {X}}$. The problems of finding a minimum supertree [18] or synteny supertree [12], respectively for single and segmental events, have been shown NP-hard for the simplest DL distance model. A naive approach consists in generating all supertrees (respec. synteny supertrees), reconcile each of them with the species tree, and keep one leading to the minimum cost. We call *NaiveSynesth* the pipeline which consists in applying Synesth to each possible supertree in turn. Here, we present *FullSynesth*, an algorithm for simultaneously building and reconciling an optimal synteny supertree, which combines the dynamic programming approach of Synesth with the supertree reconstruction strategy of [19]. In contrast to NaiveSynesth whose worst case running time is double factorial in the sum of sizes of all gene trees, FullSynesth is exponential in the number of gene families composing the syntenies, which is usually a much smaller parameter. By running the algorithms on simulated datasets, we show that FullSynesth is many orders of magnitudes faster than NaiveSynesth.

After introducing the required notations in Section 2, we define our model of evolution of syntenies in Section 3 and state the problem in Section 4. In Section 5, we show that the problem in NP-complete and then present our resolution method in Section 6. Finally, we present results on simulated datasets in Section 7.

## Notations on Trees and Syntenies

All trees considered in this paper are rooted and unordered. Given a tree *T*, we denote by *r*(*T*) its root, by *V*(*T*) its node set and by $L(T) \subseteq V(T)$ its leafset. We say that *T* is *a tree on L* if $L(T) = L$. A node $v'$ is an *ancestor* of *v* if $v'$ is on the (inclusive) path between *v* and the root, and we then call *v* a *descendant* of $v'$. The node $v'=p(v)$ immediately preceding $v \ne r(T)$ on this path is the *parent* of *v*, and then *v* is a *child* of $v'$. The ancestor–descendant relation is denoted $\le$ and forms a partial order on nodes, in which the root is minimal and the leaves are maximal. A node $v$ is a *strict ancestor* of $v'$, denoted $v < v'$, if $v \le v'$ and $v \ne v'$. Any pair of nodes $v$ and $v'$ not ordered by the $\le$ relation are said to be *separated*, which we denote $v \mathrel {\Vert }v'$. The set of children of any node $v$ is denoted by $\textrm{ch}(v)$. If $|\textrm{ch}(v)| = 1$, then $v$ is said to be *unary* and we denote its only child by $v_\textrm{c}$. If $|\textrm{ch}(v)| = 2$, then it is said to be *binary* and we arbitrarily denote its children by $v_\mathrm {\ell }$ and $v_\textrm{r}$. In that case, we say that $v_\mathrm {\ell }$ and $v_\textrm{r}$ are *siblings*. We denote by *E*(*T*) the edge set of *T*, where each edge is represented by a pair of nodes (*p*(*v*), *v*). For any two nodes $v$ and $v'$ of $T$, there exists a unique path from $v$ to $v'$ that we denote $\textrm{P}_{T}(v, v') \subseteq \textrm{E}(T)$.

We denote by $T_v$ the subtree of *T* rooted at a node *v*, i.e. obtained from *T* by removing all the nodes which are not descendants of *v*. A *binary tree* is a tree where all internal (non-leaf) nodes are binary. If all internal nodes are unary or binary, then the tree is *partially binary*. For two trees $T_1$ and $T_2$, we write $T_1 = T_2$ iff there is an isomorphism preserving leaf labels between $T_1$ and $T_2$.

A tree $T'$ is said to be an *extension* of a tree $T$ if $T'$ can be obtained from $T$ by a sequence of operations among: (1) *subdividing* an edge $(u, w)$ by adding a new node $v$ and replacing $(u, w)$ by two edges $(u, v)$ and $(v, w)$; (2) *grafting* a new node $v$ below an existing unary node $u$ by adding the edge $(u, v)$; (3) *rerooting* the tree to a new node $u$ by adding the edge $(u, \textrm{r}(T))$. If $T'$ is an extension of *T* and *v* is a node of *T*, then we denote by $\varPsi _{T'}(v)$ the node of $T'$ corresponding to *v*.

**Fig. 1:**
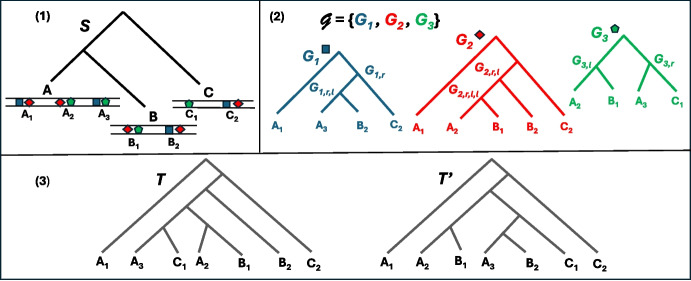
(1) A species tree $S$ on the set of species $\varSigma = \{A,B,C\}$. Each species is represented as a linear genome with syntenies on the set $\mathcal {F}$ containing the blue, red and green gene families. The set of syntenies is $\mathcal {X}= \{A_1, A_2, A_3, B_1, B_2, C_1, C_2\},$ each $X_i$ belonging to the species *X*. (2) The set $\mathcal {G}= \{G_1, G_2, G_3\}$ of the gene trees for the blue, red and green gene families. Gene copies are identified (on leaves) by the synteny each one belongs to. This set of trees is consistent. (3) Two synteny supertrees for $\mathcal {G}$

The *lowest common ancestor* (lca) of a subset $V'$ of *V*(*T*), denoted $lca_T(V')$, is the ancestor common to all nodes in $V'$ most distant from the root. The restriction $T|_{L'}$ of *T* to $L'\subseteq L(T)$, where $L' \ne \emptyset$, is the tree with leafset $L'$ obtained from the subtree of *T* rooted at $lca_T(L')$ by removing all leaves not in $L'$, and removing all internal nodes with a single child. We generalize this notation to a set of trees: For a set $\mathbb {T}$ of trees, $\mathbb {T}|_{L'} = \{T|_{L' \cap L(T)}\,: \, (T \in \mathbb {T}) \wedge (L' \cap L(T) \ne \emptyset )\}$. Let $T'$ be a tree such that $L(T') = L' \subseteq L(T)$. We say that *T* *displays* $T'$ if $T|_{L'} = T'$. A set $\mathbb {T}$ of trees is said to be *consistent* if there is a tree *T* displaying every tree of $\mathbb {T}$. A *supertree* for a set $\mathbb {T}$ of consistent trees is a tree displaying them all. See Fig. 1 for an example.

Finally, a *bipartition* of a set $L$ is a pair $\{L_1, L_2\}$ of non-empty subsets of $L$ such that $L_1 \cup L_2 = L$ and $L_1 \cap L_2 = \emptyset$. For a binary node *v* in a tree $T$, we call $\{L(T_{v_\ell }), L(T_{v_r})\}$ the bipartition of *v*.

*Species, genes and synteny trees:* A *species tree* $S$ is a binary tree on a set of species ${\varSigma }$ and a *gene tree* $G$ is a binary tree on a gene family $\varGamma$. A set of syntenies is a set of genomic segments in a set of genomes (potentially with multiple segments per genome). In this paper, each synteny contains a set of genes from a set of gene families $\mathcal {F}$, where the genes of a given synteny all belong to different gene families (i.e. repeated gene copies inside a synteny are ignored). Therefore, from now on, a gene is simply identified by the family $\varGamma \in {\mathcal {F}}$ it belongs to. Consider a set $\mathcal {X}$ of syntenies in ${\varSigma }$. A *synteny tree* *T* is a tree on $\mathcal {X}$. We will identify the leaves of the gene tree for $\varGamma$ by the unique synteny to which the corresponding gene belongs. Note that no two such leaves are identified with the same synteny. Conceptually, the leaves of a synteny tree *T* represent syntenies, while the leaves of a gene tree *G* represent genes identified by the synteny they belong to. Let $\mathcal {G}$ be a set of consistent gene trees, one for each family of $\mathcal {F}$. With the above notation (leaves of gene trees identified by the syntenies they belong to), we can consider them all as trees on ${\mathcal {X}}$. We call *synteny supertree* a supertree for $\mathcal {G}$. See an example in Fig. 1.

**Fig. 2:**
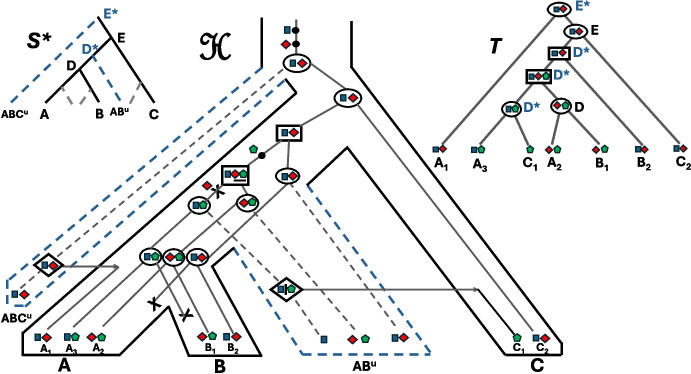
(Left): The augmented species tree $S^*$ obtained from species tree *S* in Fig. 1.(1). We use the notation of Definition 2 for the names of unsampled leaves. Moreover, for clarity, we assign names to internal nodes. The blue dotted branches of $S^*$ represent the edges leading to unsampled leaves which are used for transfers in $\mathcal {H}$. (Center): A history $\mathcal {H}$ on $S^*$ explicating the synteny supertree *T* in Fig. 1.(3), the history tree is represented with thin lines inside the species. Events are represented as follows: “$\textrm{Spe}$” by ovals, “$\textrm{Dup}$” and “$\textrm{Cut}$” by rectangles, “$\textrm{TrDup}$” and “$\textrm{TrCut}$” by diamonds, “$\textrm{Loss}$” by crosses and “$\textrm{Gain}$” by circles. The synteny contents are represented inside of each event, while the associated species is represented implicitly by the position of each event on top of the species tree. Duplicated or transferred genes are underlined, while fissions are represented by a separation in the synteny. Note that the unsampled leaves represented by grey dotted branches in $S^*$ and their parents are not represented in $\mathcal {H}$. (Right): The synteny supertree *T* with internal nodes labeled with the following information from $\mathcal {H}$: event, synteny content, and associated species from $S^*$

For a synteny or gene tree *T*, we define $x: L(T) \rightarrow \mathcal {P}(\mathcal {F})$ the function mapping each synteny to its *content* which is the set of gene families that appear in the synteny, and $s: L(T) \rightarrow \varSigma$ the function mapping each synteny to its corresponding species. We say that a set $\mathcal {G}$ of trees *is on* ${\mathcal {X}}$ if $\cup _{G \in \mathcal {G}}L(G) = {\mathcal {X}}$. For a set $\mathcal {G}$ of trees on ${\mathcal {X}}$, we denote $x(\mathcal {G}) = \cup _{\xi \in {\mathcal {X}}}x(\xi )$.

Finally, the restriction of any function *f* to a subset *A* of its domain is denoted by $f|_{A}$. Moreover, we use the Iverson bracket notation $\llbracket P \rrbracket$, where $P$ is a boolean statement, and $\llbracket P \rrbracket = 1$ if $P$ is true and $\llbracket P \rrbracket = 0$ otherwise.

## Evolutionary Histories for Syntenies

We consider the evolutionary model for syntenies defined in [14], which includes divergence following speciation (“$\textrm{Spe}$”), duplication of a synteny subset (“$\textrm{Dup}$”), fission of a synteny (“$\textrm{Cut}$”), transfer of a duplicated or cut subset (resp. “$\textrm{TrDup}$” or “$\textrm{TrCut}$”), and gain or loss of a subset (resp. “$\textrm{Gain}$” or “$\textrm{Loss}$”). Losses can be partial in the sense that only a strict subset of a synteny may be lost. Evolutionary histories are sequences of such events, as formally defined below. See Fig. 2 for an example.

### Definition 1

**(Evolutionary history for syntenies)** A history $\mathcal {H}$ on a binary species tree $S$ is a tuple $\langle H, e, x, s\rangle$, where $H$ is a partially binary tree. Each node $v \in \textrm{V}(H)$ is labeled with a species $s(v) \in \textrm{V}(S)$ and a synteny content $x(v)$. Each internal node is additionally labeled with an event $e(v) \in \mathcal {E}= \{\textrm{Spe},\textrm{Dup},\textrm{Cut},\textrm{TrDup},\textrm{TrCut},\textrm{Gain},\textrm{Loss}\}$ acting on $x(v)$ and $s(v)$. These labelings satisfy the following conditions:

1. labeldef:histspsspe If $e(v) = \textrm{Spe}$, then *v* is binary; let $\textrm{ch}(v) = \{v', v''\}$ and $\sigma = s(v)$, then $x(v) = x(v') = x(v'')$ and $\{s(v'), s(v'')\} = \textrm{ch}(\sigma )$.
2. If $e(v) \in \{\textrm{Dup}, \textrm{Cut}, \textrm{TrDup}, \textrm{TrCut}\}$, then *v* is binary; let $\textrm{ch}(v) = \{v_\textrm{t}, v_\textrm{k}\}$:
  1. if $$e(v) \in \{\textrm{Dup}, \textrm{TrDup}\}$$, then $$x(v_\textrm{t}) \subseteq x(v) = x(v_\textrm{k})$$;
  2. if $e(v) \in \{\textrm{Cut}, \textrm{TrCut}\}$, then $x(v_\textrm{t}) \cup x(v_\textrm{k}) = x(v)$ and $x(v_\textrm{t}) \cap x(v_\textrm{k}) = \emptyset$ (but $x(v_\textrm{t}) = \emptyset$ or $x(v_\textrm{k}) = \emptyset$ is allowed and in this case the cut is *full*);
  3. if $$e(v) \in \{\textrm{Dup}, \textrm{Cut}\}$$, then $$s(v) = s(v_\textrm{t}) = s(v_\textrm{k})$$;
  4. if $e(v) \in \{\textrm{TrDup}, \textrm{TrCut}\}$, then $s(v) \mathrel {\Vert }s(v_\textrm{t})$, and $s(v) = s(v_\textrm{k})$.
3. If $e(v) \in \{\textrm{Gain}, \textrm{Loss}\}$, then *v* is unary; let $\textrm{ch}(v) = \{v_\textrm{c}\}$, then $s(v_\textrm{c}) = s(v)$ and:
  1. if $$e(v) = \textrm{Gain}$$, then $$x(v_\textrm{c}) \supsetneq x(v)$$.
  2. if $e(v) = \textrm{Loss}$, then $x(v_\textrm{c}) \subsetneq x(v)$ (a loss is *full* if $x(v_\textrm{c}) = \emptyset$, and *partial* otherwise);
4. For each gene family $\varGamma$, exactly one $\textrm{Gain}$ event in $H$ involves $\varGamma$.

Condition (4) excludes *convergent gains*, i.e. histories where the same gene family is gained separately in different parts of the history. Moreover, as in [14], we allow for transfers from unsampled species by augmenting the species tree.

### Definition 2

**(Augmented species tree)** A tree $S$ on a set of species $\varSigma$ can be augmented into a tree $S^*$ on a set $\varSigma ^* \supseteq \varSigma$ by grafting *unsampled leaves* as follows: (1) subdivide each edge $(v, v')$ of $\textrm{E}(S)$ into two edges $(v, z)$ and $(z, v')$ through a new node $z$, (2) connect each $z$ to a new unsampled leaf labeled by $L^{\text {u}} \in \varSigma ^* - \varSigma$ where $L$ is the set such that $v' = \textrm{lca}_{S}(L)$, and (3) create a new root $\textrm{r}(S^*)$ whose two children are $\textrm{r}(S)$ and a new unsampled leaf labeled by $\varSigma ^{\text {u}}$. A node $\sigma \in V(S^*)$ which is not an unsampled leaf is said to be *sampled*.

### Definition 3

**(Visible leaves)** A leaf $l$ of a history $\mathcal {H}= \langle H, e, x, s\rangle$ is said to be *visible* if $x(l) \ne \emptyset$ and $s(l)$ is sampled, and *invisible* otherwise. The set of visible leaves of $\mathcal {H}$ is denoted $\textrm{L}_V(\mathcal {H})$.

### Definition 4

**(Explicatory histories)** For a species tree $S$, a history $\mathcal {H}= \langle H, e, x', s'\rangle$ on $S^*$ is said to *explicate* $S$ and $\langle T, x, s\rangle$, a synteny tree *T* with its associated mappings $x$ and $s$, if:

1. $H$ is an extension of $T$.
2. $$\forall \; v \in L(T)$$, $$x'(\varPsi _H(v)) = x(v)$$ and $$s'(\varPsi _H(v)) = s(v)$$.
3. $$\textrm{L}_V(\mathcal {H}) = \{\varPsi _H(v) \mid v \in \textrm{L}(T)\}$$.
4. No $(u,v) \in \textrm{E}(T)$ is such that $s(\varPsi _H(v)) < s(\varPsi _H(u))$.

The set of all such histories is denoted by $\mathbb {H}(T, S)$.

Condition (4) excludes assignments of species which create cycles between adjacent nodes of the synteny tree. This condition is a necessary, but not sufficient, condition for acyclicity. Imposing a full acyclicity condition would lead to computationally-intractable problems [3].

If *T* is a synteny supertree for a set of consistent gene trees $\mathcal {G}$, then any node *v* of a tree in $\mathcal {G}$ corresponds to a node of *T* which itself corresponds to a node of *H* denoted $\varPsi _H(v)$.

## Problem Statement

The cost of a history will depend on the number of events of each type. As in [14], for simplicity, we disregard the number of gains. As usual for reconciliation, we also ignore the number of speciations, since they do not allow to meaningfully distinguish histories.

### Definition 5

**(Event vector)** Let $\mathcal {H}= \langle H, e, x, s\rangle$ be a history. We define $\textrm{ev}(\mathcal {H}) = (c_{\textrm{Dup}},c_{\textrm{Cut}},c_{\textrm{TrDup}},c_{\textrm{TrCut}},c_{\textrm{Loss}}) \in \mathbb {N}^5$ as the vector such that $c_{\textit{ev}}$ is the number of events of type $\textit{ev} \in \mathcal {E}$ in $\mathcal {H}$.

Given an event cost vector $\delta = (\delta _\textrm{Dup},\delta _\textrm{Cut},\delta _\textrm{TrDup},\delta _\textrm{TrCut},\delta _\textrm{Loss})$ such that $\delta \in (\mathbb {R}_{\ge 0} \cup \{\infty \})^5$, we can associate an overall scalar cost $\textrm{c}(\mathcal {H}) = \delta \cdot \textrm{ev}(\mathcal {H})$ to each history.

For a set $\mathcal {G} = \{G_1, G_2, \dots , G_k\}$ of consistent gene trees on ${\mathcal {X}}$, we denote by $\mathbb {T}(\mathcal {G})$ the set of all binary synteny supertrees of $\mathcal {G}$ on ${\mathcal {X}}$. Moreover, for a synteny supertree $T$ and a species tree $S$, we denote $c^{\textrm{min}}(T, S) = \min \{ \textrm{c}(\mathcal {H}) \mid \mathcal {H}\in \mathbb {H}(T, S) \}$. We are now ready to set our problem which consists in simultaneously building and reconciling a synteny supertree.

Minimum synteny supertree Problem (MinSynSupertree):

**Input:** A species tree *S* on ${\varSigma }$, a set ${\mathcal {X}}$ of syntenies in ${\varSigma }$, a set $\mathcal {G}= \{G_1,\dots , G_k\}$ of consistent gene trees on ${\mathcal {X}}$ and a cost vector $\delta$.

**Output:** $$ \min _{T \in \mathbb {T}(\mathcal {G})} \{c^{\textrm{min}}(T, S)\}$$.

## Complexity

We show in this section that the decision version of the MinSynSupertree Problem is NP-Complete for a unitary cost, i.e. when $\delta = (1,1,1,1,1)$. The decision version of the MinSynSupertree Problem is defined as follows.

Minimum synteny supertree decision Problem

(MinSynSupertreeDecision):

**Input:** A species tree *S* on ${\varSigma }$, a set ${\mathcal {X}}$ of syntenies in ${\varSigma }$, a set $\mathcal {G}= \{G_1,\dots , G_k\}$ of consistent gene trees on ${\mathcal {X}}$, a cost vector $\delta$, and a positive integer *b*.

**Question:** Is there a tree $T \in \mathbb {T}(\mathcal {G})$ such that $c^{\textrm{min}}(T, S) \le b$?

Note that given a tree $T \in \mathbb {T}(\mathcal {G})$, $c^{\textrm{min}}(T, S)$ can be computed in polynomial time [14] and therefore MinSynSupertreeDecision is in NP.

We now show that MinSynSupertreeDecision is NP-hard by reduction from the Monotone one-in-three-satisfiability problem (Monotone 1-in-3-SAT Problem), defined as follows (monotone meaning that there are no negation of variables in the clauses).

Monotone 1-in-3-SAT Problem:

**Input:** A formula ${\mathcal {C}}= (C_1 \wedge C_2 \wedge \dots \wedge C_k)$ on a finite set $\mathcal {L} = \{\ell _1,\ell _2,\dots ,\ell _m\}$ of variables where each $C_i$, $1 \le i \le k$, is a clause of the form $(x \vee y \vee z)$ with $\{x,y,z\} \subseteq \mathcal {L}$ and *x*, *y* and *z* distinct.

**Question:** Is there a truth assignment such that exactly one literal in each clause of *C* is set to *True*?

The Monotone 1-in-3-SAT Problem is NP-Complete by Schaefer’s dichotomy theorem [20].

Given an instance ${\mathcal {I}}= ({\mathcal {C}},\mathcal {L})$ of the Monotone 1-in-3-SAT problem, we construct, in polynomial time, a corresponding instance ${\mathcal {I}}' = (S,{\mathcal {X}},\mathcal {G},\delta ,b)$ of the MinSynSupertreeDecision problem.

**Fig. 3:**
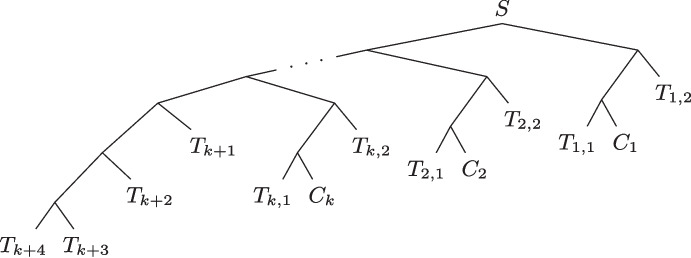
The species tree $S$ on $\varSigma$

The set of extant species $\varSigma$ contains the species $C_i$, $T_{i,1}$, and $T_{i,2}$ for each $i \in \{1,2,\dots ,k\}$ and also the four species $T_{k+1}$, $T_{k+2}$, $T_{k+3}$ and $T_{k+4}$. The species tree $S$ on $\varSigma$ is represented in Fig. 3.

In the following, we say that a given synteny *v* is mapped to a species $\sigma$ if $s(v) = \sigma$. The set of syntenies ${\mathcal {X}}$ is then defined as follows:

- For $i \in \{1,2,\dots ,k\}$ and $y \in \{1,2\}$, ${\mathcal {X}}$ contains:
  - The syntenies $T_{i,y,1}$, $T_{i,y,2}$, $T_{i,y,3}$, $T'_{i,y,1}$, $T'_{i,y,2}$ and $T'_{i,y,3}$ mapped to the species $T_{i,y}$.
  - The syntenies $C_{i,1}$, $C_{i,2}$ and $C_{i,3}$ mapped to the species $C_i$.
- For $x \in \{k+1,k+2,k+3,k+4\}$ and $y \in \{1,2\}$, ${\mathcal {X}}$ contains:
  - The syntenies $T_{x,y,1}$, $T_{x,y,2}$, $T_{x,y,3}$, $T'_{x,y,1}$, $T'_{x,y,2}$ and $T'_{x,y,3}$ mapped to the species $T_{x}$.

The gene family contents of the syntenies in ${\mathcal {X}}$ are determined by the gene trees they appear in: each gene tree in $\mathcal {G}$ (defined below) corresponds to a distinct gene family and each synteny in ${\mathcal {X}}$ contains the gene families corresponding to the gene trees they appear in. The set of all gene families is noted $\mathcal {F}$.

**Fig. 4:**
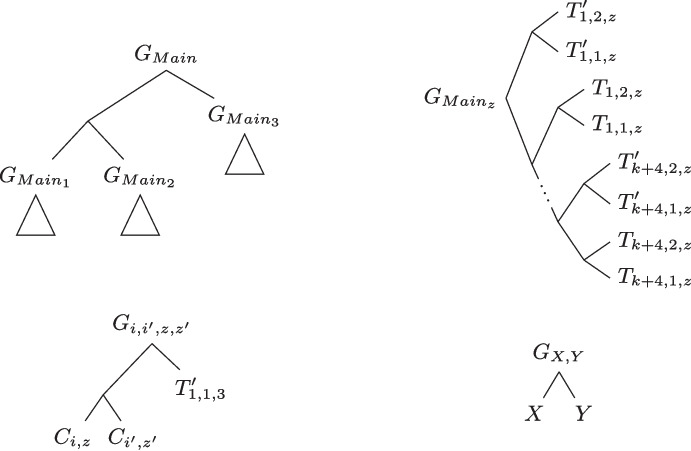
The elements of $\mathcal {G}$. *(Top-left panel:)* The gene tree $G_{Main}$ is made up of the trees $G_{Main_z}$ for $z \in \{1,2,3\}$. *(Top-right panel:)* Shape of one of the $G_{Main_z}$ subtrees of $G_{Main}$. *(Bottom-left panel:)* A gene tree $G_{i,i',z,z'}$, element of $\mathcal {G}_{Variables}$. *(Bottom-right panel:)* A gene tree $G_{X,Y}$, element of $\mathcal {G}_{Syntenies}$

We now detail the gene tree set $\mathcal {G}$ on ${\mathcal {X}}$. This set is made up of three parts, $\mathcal {G} = \{G_{Main}\} \cup \mathcal {G}_{Variables} \cup \mathcal {G}_{Syntenies}$, represented in Fig. 4. The set of trees $\mathcal {G}_{Variables}$ contains a tree $G_{i,i',z,z'}$ for each $i,i',z,z'$ ($1 \le i,i' \le k$ and $z,z' \in \{1,2,3\}$) such that the $z$^th^ literal of the clause $C_i$ is the same as the $z'$^th^ literal of the clause $C_i'$. The set of trees $\mathcal {G}_{Syntenies}$ contains a tree $G_{X,Y}$ for each $X,Y \in {\mathcal {X}}$ such that $X \ne Y$.

Note that $\mathcal {G}$ is a consistent set of gene trees, because we may construct a supertree displaying all trees in $\mathcal {G}$ by starting from the tree $\mathcal {G}_{Main}$ and then graft the syntenies $C_{i,z}$ ($1 \le i \le k$, $z \in \{1,2,3\}$) in the $G_{Main_1}$ subtree of $G_{Main}$ (after subdividing some edges in the subtree).

Finally, we set $\delta = (1,1,1,1,1)$ and $b = 50 + 15k$.

We next show that ${\mathcal {I}}$ is a satisfiable instance of the Monotone 1-in-3-SAT problem if and only if (Lemmas 1 and 2) its corresponding instance ${\mathcal {I}}'$ of the MinSynSupertreeDecision problem admits a tree $T \in \mathbb {T}(\mathcal {G})$ such that $c^{\textrm{min}}(T, S) \le b$.

The idea of the proof is that we can construct a tree $T \in \mathbb {T}(\mathcal {G})$ by subdividing 3*k* edges in $G_{Main}$ and then graft the syntenies $C_{i,z}$ ($1 \le i \le k$, $z \in \{1,2,3\}$) below the 3*k* newly added nodes. These syntenies represent the litterals in the clauses and for each clause $C_i$, exactly one of $C_{i,1}$, $C_{i,2}$ and $C_{i,3}$ will be grafted in each of the subtrees $T_{\varPsi _T(r(G_{Main_1}))}$, $T_{\varPsi _T(r(G_{Main_2}))}$ and $T_{\varPsi _T(r(G_{Main_3}))}$. The litteral corresponding to the synteny grafted in the subtree $T_{\varPsi _T(r(G_{Main_3}))}$ is then set to *True* in the clause $C_i$ and the two others are set to *False*.

### Lemma 1

Let ${\mathcal {I}}$ be a satisfiable instance of the Monotone 1-in-3-SAT problem. Then its corresponding instance ${\mathcal {I}}'$ of the MinSynSupertreeDecision problem admits a tree $T \in \mathbb {T}(\mathcal {G})$ such that $c^{\textrm{min}}(T, S) \le 50 + 15k$.

### Proof

Notice that the only syntenies of ${\mathcal {X}}$ missing from the tree $G_{Main}$ are the syntenies $C_{i,z}$ ($1 \le i \le k$, $z \in \{1,2,3\}$). We can thus construct a tree $T \in \mathbb {T}(\mathcal {G})$ by subdividing 3*k* edges in $G_{Main}$ and then graft those 3*k* syntenies below the 3*k* newly added nodes.

Let *TA* be a truth assignment satisfying ${\mathcal {C}}$ such that exactly one literal in each clause is set to *True* (we know that such truth assignment exists because ${\mathcal {I}}$ is a satisfiable instance). Without loss of generality, we will assume that the third literal of each clause is the one set to *True*.

**Fig. 5:**
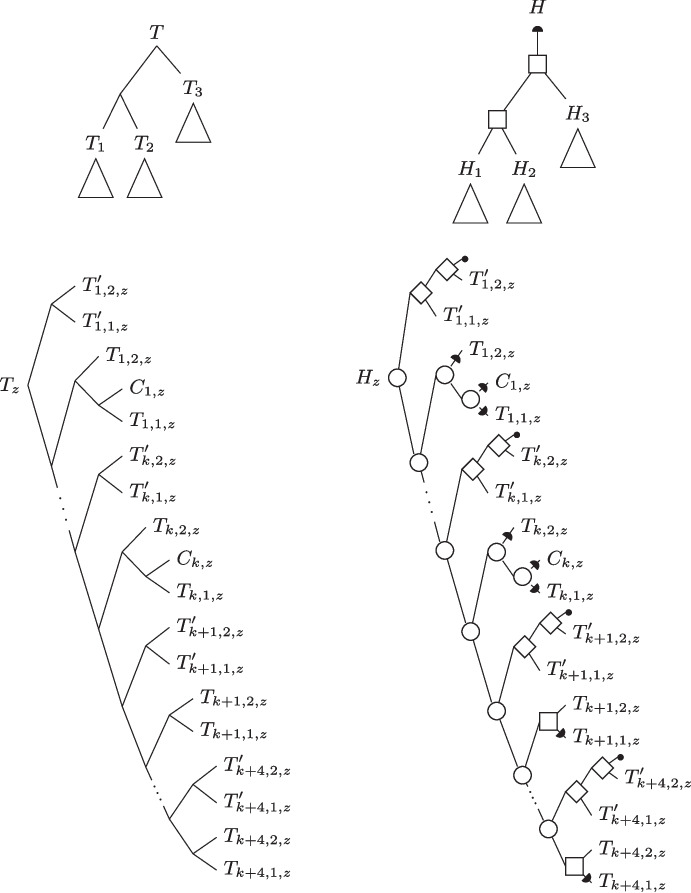
*(Left panel:)* The synteny tree $T$, which is an extension of the $G_{Main}$ tree, is made up of the trees $T_z$ for $z \in \{1, 2, 3\}$. *(Right panel:)* The history tree $H$ is made up of the trees $H_z$ for $z \in \{1,2,3\}$. A circle indicates a $\textrm{Spe}$, a square indicates a $\textrm{Dup}$, a diamond indicates a $\textrm{TrDup}$, an upper half-circle indicates a $\textrm{Gain}$, and a lower half-circle indicates a $\textrm{Loss}$. Terminal filled circles indicate leaves in unsampled species. For brevity, speciations leading to unsampled leaves are not shown in this figure

We can construct $T\in \mathbb {T}(\mathcal {G})$ (see Fig. 5, left panel) such that $c^{\textrm{min}}(T, S) \le 50 + 15k$ as follows:

- Initialize *T* = $$G_{Main}$$.
- For each *i*, $1 \le i \le k$, we subdivide the edge $(p(T_{i,1,3}),T_{i,1,3})$ of *T* and graft the synteny $C_{i,3}$ below the newly added node. Then, we subdivide the edges $(p(T_{i,1,1}),T_{i,1,1})$ and $(p(T_{i,1,2}),T_{i,1,2})$ and graft the other two syntenies mapped to the species $C_i$ below the newly added nodes (it doesn’t matter which synteny is grafted to which edge).

Note that *T* displays $G_{Main}$, and the trees in $\mathcal {G}_{Syntenies}$ by construction. Furthermore, *T* displays all trees in $\mathcal {G}_{Variables}$ because if there are $i,i',z,z'$ ($1 \le i,i' \le k$ and $z,z' \in \{1,2,3\}$) such that the *z*^th^ literal of the clause $C_i$ is the same as the $z'$^th^ literal of the clause $C_i'$, then the syntenies $C_{i,z}$ and $C_{i',z'}$ are both grafted in the subtree of *T* rooted at $\varPsi _T(r(G_{Main_3})_\ell )$ if the corresponding literal is set to *True* in *TA* and they are both grafted in the subtree rooted at $\varPsi _T(p(r(G_{Main_1})))$ otherwise by construction. Thus, $T \in \mathbb {T}(\mathcal {G})$. Recall that $S^*$ is obtained from *S* (in Fig. 3) by grafting an unsampled leaf on each edge and adding a new root with an unsampled leaf as a child and the root of *S* as the other. We can obtain a history $\mathcal {H}= \langle H, e, x, s\rangle$ (see Fig. 5, right panel) explicating *T* and *S* of cost $50 + 15k$ as follows:

- Initialize $H = T$ and set $s(r(H)) = r(S^*)$.
- Set $$e(r(H)) = \textrm{Dup}$$ and $$e(r(H)_\ell ) = \textrm{Dup}$$.
- Set $e(p(T_{x,1,z})) = \textrm{Dup}$ for all $x \in \{k+1,k+2,k+3,k+4\}$ and $z \in \{1,2,3\}$.
- Add a partial loss node between the nodes of the edge $(p(T_{x,1,z}),T_{x,1,z})$ (by subdividing the edge) for all $x \in \{k+1,k+2,k+3,k+4\}$ and $z \in \{1,2,3\}$.
- Set $e(p(T'_{x,1,z})) = \textrm{TrDup}$ transferring to the right side for all $x \in \{1,2,\dots ,k+4\}$ and $z \in \{1,2,3\}$.
- Add a $\textrm{TrDup}$ event between the nodes of the edge $(p(T'_{x,1,z}),T'_{x,1,z})$ (by subdividing the edge) with one child being $T'_{x,1,z}$ in the direction of the transfer and the other an unsampled leaf for all $x \in \{1,2,\dots ,k+4\}$ and $z \in \{1,2,3\}$.
- For all $i \in \{1,2,\dots ,k\}$ and $z \in \{1,2,3\}$, subdivide and then graft an unsampled leave on the edges $(p(C_{i,z}),C_{i,z})$, $(p(T_{i,1,z}),T_{i,1,z})$, $(p(T_{i,2,z}),T_{i,2,z})$, $(p(p(C_{i,z})),p(C_{i,z}))$, $(p(p(p(C_{i,z}))),p(p(C_{i,z})))$. For all $x \in \{k+1, k+2, k+3\}$ and $z \in \{1,2,3\}$, subdivide and then graft an unsampled leave on the edge $(p(p(T_{x,2,z})),p(T_{x,2,z}))$. Note that these nodes are not shown in Fig. 5 for brevity.
- Add partial losses between the nodes of the edges $(p(T_{i,1,z}),T_{i,1,z})$, $(p(T_{i,2,z}),T_{i,2,z})$ and $(p(C_{i,z}),C_{i,z})$ for all $i \in \{1,2,\dots ,k\}$ and $z \in \{1,2,3\}$.
- Set $e(v) = \textrm{Spe}$ for each internal node $v \in V(H)$ that has not yet been assigned an event.
- Set *s*(*v*) for each internal node *v* of *H* corresponding to a node in *T* following the events in the history, starting from the root ($s(r(H)) = r(S^*)$).
- Create a new root *r* for *H* such that *r* is an unary node, $s(r) = r(S^*)$, $x(r) = \emptyset$ and $e(r) = \textrm{Gain}$. Set $x(v) = \mathcal {F}$ for all other internal nodes $v \in V(H)$.

Note that $\mathcal {H}$ is a valid history explicating *T* because the species at each node of the history respects the definition of the events by construction and for each leaf *v* of *T*, there is a distinct event on the path from $\varPsi _H(p(v))$ to $\varPsi _H(v)$ allowing to lose the required content. As there are 14 $\textrm{Dup}$, 0 $\textrm{Cut}$, $24+6k$ $\textrm{TrDup}$, 0 $\textrm{TrCut}$ and $12 + 9k$ $\textrm{Loss}$ in $\mathcal {H}$, $c(\mathcal {H}) = 50+15k$. Therefore, $c^{\textrm{min}}(T, S) \le 50 + 15k$. $\square$

### Lemma 2

Let ${\mathcal {I}}'$ be an instance of the MinSynSupertreeDecision problem admitting a tree $T \in \mathbb {T}(\mathcal {G})$ such that $c^{\textrm{min}}(T, S) \le 50+15k$. Then its corresponding instance ${\mathcal {I}}$ of the Monotone 1-in-3-SAT problem is satisfiable.

### Proof

Let *T* be a synteny tree in $\mathbb {T}(\mathcal {G})$ such that $c^{\textrm{min}}(T, S) \le 50+15k$ and let $\mathcal {H}= \langle H, e, x, s\rangle$ be a history explicating *T* of minimum cost. We will show that *T* and H have the same forms as the trees *T* and *H* in Fig. 5.

We will then show that we can obtain a truth assignment *TA* satisfying ${\mathcal {C}}$ by setting $\ell _j$, $1 \le j \le m$, to *True* if there is at least one synteny $C_{i,z}$ ($1 \le i \le k$, $z \in \{1,2,3\}$) that is a descendant of $\varPsi _T(r(G_{Main_3}))$ such that the *z*^th^ literal of the clause $C_i$ is $\ell _j$ and *False* otherwise.

We first show that for each clause $C_i$ ($1 \le i \le k$), there is exactly one value $z \in \{1,2,3\}$ such that $C_{i,z}$ is a descendant of $\varPsi _T(r(G_{Main_3}))$.

First notice that for every pair of distinct leaves $v_1$ and $v_2$ of *T*, $x(v_1) \ne x(v_2)$ and $x(v_1) \cap x(v_2)$ contains a gene family not present in the content of any other leaf of *T* by construction. Thus, for each leaf *v* of *T* there must be an event on the path from $\varPsi _H(p(v))$ to $\varPsi _H(v)$ allowing to lose some synteny content because, by definition of a history, $x(\varPsi _H(p(v)))$ must contain some of the content of the sibling of *v* (the content that the sibling of *v* shares with any other leaves than *v*) which differs from the content of *v*. Note that $e(\varPsi _H(p(v))) \notin \{\textrm{Cut},\textrm{TrCut}\}$ because the intersection between the content of *v* and its sibling’s content is not empty and thus the event on the path from $\varPsi _H(p(v))$ to $\varPsi _H(v)$ required for the leaf *v* is distinct from the event required for any other leaf of *T*. Therefore, as there are $48+15k$ leaves in *T* by construction, $c^{\textrm{min}}(T, S) \ge 48+15k$ and at most two nodes of *H* corresponding to nodes of *T* that are not parent of a leaf may be assigned to an event other than speciation because otherwise $c^{\textrm{min}}(T, S)$ would be greater than $50+15k$.

We will now show that the nodes $\varPsi _H(r(G_{Main}))$ and $\varPsi _H(r(G_{Main})_{\ell })$ cannot be assigned to speciations. Let $S^*$ be the augmented species tree of *S*. Notice that the longest path from the root of $S^*$ to a leaf of $S^*$ is the path from $r(S^*)$ to $\varPsi _{S^*}(T_{K+4})$ of length $2(k+3)+1$. Thus, there cannot be a path in *H* from an ancestor to a descendant containing more than $2(k+3)+1$ speciation nodes if no transfer event switches the species along the way, and this can happen only if the root of the path is mapped to $r(S^*)$. Therefore, the only way for all the nodes in the paths from $\varPsi _H(r(G_{Main_z}))$ to $\varPsi _H(p(T_{k+4,1,z}))$ ($z \in \{1,2,3\}$) which are not parents of visible leaves (we can ignore the parents of a leaf $C_{i,z}$ that would be grafted onto the path because such nodes cannot change the species in the path) to be assigned to speciations is if $s(\varPsi _H(r(G_{Main_z}))) = r(S^*)$ because those paths are of length $2(k+3)+1$ (without accounting for grafted leaves $C_{i,z}$). Let us now assume, for the sake of contradiction, that $e(\varPsi _H(r(G_{Main})))=\textrm{Spe}$. In that case, the nodes $\varPsi _H(r(G_{Main_z}))$ ($z \in \{1,2,3\}$) cannot be mapped to the root of $S^*$ (because no transfer event may transfer to $r(S^*)$, since it is an ancestor of every node in $S^*$) and thus $c^{\textrm{min}}(T, S) > 50+15k$ which is a contradiction. Therefore, $e(\varPsi _H(r(G_{Main}))) \ne \textrm{Spe}$ and $c^{\textrm{min}}(T, S) \ge 49+15k$. Using a similar argument, we may conclude that $\varPsi _H(r(G_{Main})_{\ell })$ can’t be assigned to a speciation node either, as otherwise $c^{\textrm{min}}(T, S)$ would be greater than $50+15k$.

We now show that $c^{\textrm{min}}(T, S) \le 50+15k$ if and only if for every subtree $T_{\varPsi _T(p(T_{i,2,z}))}$ ($1 \le i \le k$ and $z \in \{1,2,3\}$) there is exactly one leaf mapped to $C_i$ in the subtree. As $\varPsi _H(r(G_{Main}))$ and $\varPsi _H(r(G_{Main})_{\ell })$ are not assigned to speciations, $c^{\textrm{min}}(T, S)\ge 50+15k$ and the only way for $c^{\textrm{min}}(T, S)$ to be equal to $50+15k$ is if every other binary node in *H* which is not parent of a leaf is assigned to a speciation event. In particular, this means that every node on the path from $\varPsi _H(r(G_{Main_z}))$ to $\varPsi _H(p(T_{k+4,1,z}))$ ($z \in \{1,2,3\}$) which is not parent of a visible leaf is assigned to a speciation event and in this case $s(\varPsi _H(r(G_{Main_z}))) = r(S^*)$ as shown earlier. Thus, $s(\varPsi _H(p(T_{i,2,z})))$ is an ancestor of $p(T_{i,2})$ as otherwise there would be an event in $\{\textrm{TrDup},\textrm{TrCut}\}$ on the path from $\varPsi _H(p(p(T_{i,2,z})))$ to $\varPsi _H(p(T_{i,2,z}))$ that is not ancestor of a visible leaf and/or there would be full losses in the history and $c^{\textrm{min}}(T, S)$ would be greater than $50+15k$. Let’s first consider the case where the only two leaves in the subtree $T_{\varPsi _T(p(T_{i,2,z}))}$ (for a given $1 \le i \le k$ and $z \in \{1,2,3\}$) are $\varPsi _T(T_{i,1,z})$ and $\varPsi _T(T_{i,2,z})$. In that case, the cost of the subhistory rooted at $\varPsi _H(p(T_{i,2,z}))$ is at least 3 (because 2 events are required for the content of the two leaves and getting to the species $s(T_{1,1,z})$ from an ancestor of $p(T_{i,2})$ requires at least one other event) and thus $c^{\textrm{min}}(T, S)$ is greater than $50+15k$ which is a contradiction. Therefore, the only way for $c^{\textrm{min}}(T, S)$ to be less than or equal to $50+15k$ is if there is at least one leaf grafted in each subtree $T_{\varPsi _T(p(T_{i,2,z}))}$ for $1 \le i \le k$ and $z \in \{1,2,3\}$. As there are 3*k* such subtrees and exactly 3*k* leaves to be grafted on $G_{Main}$ to obtain *T*, we can deduce that exactly one leaf will be grafted in each subtree. We now show that the leaf grafted in the subtree $T_{\varPsi _T(p(T_{i,2,z}))}$ (for a given $1 \le i \le k$ and $z \in \{1,2,3\}$) must be mapped to $C_i$. Let’s suppose, for the sake of contradiction, that this is not the case, i.e. that there is a subtree $T_{\varPsi _T(p(T_{i,2,z}))}$ for which the leaf grafted in the subtree is mapped to $C_j$ with $j \ne i$. In that case, it is easy the see that the cost of the subhistory rooted at $\varPsi _H(p(T_{i,2,z}))$ is at least 4 because 3 events are required for the content of the 3 leaves, plus one other event, to get to the species $s(T_{1,1,z})$ from an ancestor of $p(T_{i,2})$. Thus, $c^{\textrm{min}}(T, S)$ is greater than $50+15k$, which is a contradiction. We conclude that there is a leaf mapped to $C_i$ in the subtree $T_{\varPsi _T(p(T_{i,2,z}))}$ for $1 \le i \le k$ and $z \in \{1,2,3\}$. Therefore, for each clause $C_i$ ($1 \le i \le k$), there is exactly one value $z \in \{1,2,3\}$ such that $C_{iz}$ is a descendant of $\varPsi _T(r(G_{Main_3}))$.

Finally, note that for each $i,i',z,z'$ ($1 \le i,i' \le k$, $z,z' \in \{1,2,3\}$) such that the *z*^th^ literal of the clause $C_i$ is the same as the $z'$^th^ literal of the clause $C_i'$, both $C_{iz}$ and $C_{i'z'}$ are either descendants of $\varPsi _T(r(G_{Main_3}))$ or both are not descendant of $\varPsi _T(r(G_{Main_3}))$ as otherwise *T* would not display the tree $G_{i,i',z,z'}$ which would contradict the fact that $T \in \mathbb {T}(\mathcal {G})$.

It follows that ${\mathcal {C}}$ is satisfied by *TA*. $\square$

Lemmas 1 and 2 lead to the following result.

### Theorem 1

The MinSynSupertreeDecision problem is NP-complete.

## Method

We describe, in this section, the different parts of our dynamic programming algorithm *FullSynesth* for solving MinSynSupertree. For conciseness, the described algorithm simply outputs the minimum cost. However, it can easily be modified to also output the corresponding synteny supertree *T* and a corresponding history $\mathcal {H}$ by keeping track of the synteny tree and history corresponding to the minimum cost at each step. A pseudocode is given in Section 6.1. Its correctness follows from a series of theorems (Theorems 2 to 6) that we successively state. They lead to the time and space complexity result stated in Theorem 7.

In the following, we consider as input a species tree *S* for a set of species $\varSigma$, an augmented species tree $S^*$, a set $\mathcal {F}$ of gene families, a set ${\mathcal {X}}^{input}$ of syntenies in $\varSigma$, a set $\mathcal {G}^{input} = \{G^{input}_1, \dots , G^{input}_k\}$ on ${\mathcal {X}}^{input}$ of consistent gene trees such that $x(\mathcal {G}^{input}) =\mathcal {F}$ and an event cost vector $\delta$. Moreover, we will need a dummy gene family $\varGamma ^* \notin \mathcal {F}$ for efficient computation of synteny contents.

### Bipartitions

**Fig. 6:**
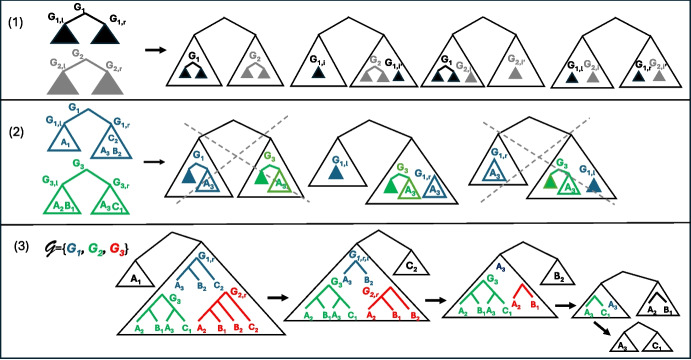
(1) The procedure for displaying all the compatible bipartitions for two consistent trees $G_1$ and $G_2$. Each bipartition is obtained by “sending” ${\mathcal {X}}_1 \in \{L(G_1), L(G_{1,l}), L(G_{1,r}), \emptyset \}$ in the left part, and the complement $L(G_1) - {\mathcal {X}}_1$ in the right part. The same process is then applied to $G_2$. In the figure, $(i,i') \in \{(l,r), (r,l)\}$, leading to seven possible splits. (2) Splits do not necessarily lead to bipartitions as illustrated on the trees $G_1$ and $G_3$ of Fig. 1. (3) A sequence of bipartitions for the set $\mathcal {G}= \{G_1, G_2, G_3\}$ leading to the synteny supertree *T* in Fig. 1. This figure was adapted and expanded from [19, Figure 2]

We take advantage of the strategy considered in [19] for the classical reconciliation model with DL distance, the difference here being the considered evolutionary model and the way the cost is recursively computed. An optimal synteny supertree *T* is constructed from the root to the leaves. At each step, i.e. for each internal node *v* being constructed in *T*, each possible bipartition $\{L(T_{v_\ell }), L(T_{v_r})\}$ that could be induced by *v* is tried, and the algorithm continues on each of $L(T_{v_\ell })$ and $L(T_{v_r})$. For example, at the root, the goal is to find a best bipartition of $\mathcal {X}^{input}$, i.e. one leading to the minimum cost. Figure 6.(3) illustrates the recurrence for reconstructing a synteny supertree. Each step of the recurrence (each triangle in Fig. 6.(3)) involves a tree set $\mathcal {G}= \mathcal {G}^{input}|_{\mathcal {X}}$ for a set of leaves ${\mathcal {X}}\subseteq {\mathcal {X}}^{input}$. We call such a set *a restriction of* $\mathcal {G}^{input}$. From now on, all considered tree sets are restrictions of $\mathcal {G}^{input}$.

One of the key ideas behind the proof of Theorem 7 on time complexity is that the constraint of displaying the input gene trees induces a strong constraint on the bipartitions. In fact, only *compatible bipartitions*, as defined below, have to be tested.

#### Definition 6

**(Compatible bipartition)** Let $\mathcal {G}= \{G_1,\ldots ,G_k\}$ be a tree set on ${\mathcal {X}}$. A bipartition $\{ {\mathcal {X}}_\ell ,{\mathcal {X}}_r \}$ of ${\mathcal {X}}$ is *compatible* with $\mathcal {G}$ (or simply “compatible” if no ambiguity on the set of leaves) if there exists a supertree of $\mathcal {G}$ such that the bipartition of its root is $\{ {\mathcal {X}}_\ell ,{\mathcal {X}}_r \}$. We denote by $\mathcal {B}(\mathcal {G})$ the set of all compatible bipartitions.

As shown in [19, Lemma 2], there are $O(4^{|\mathcal {G}|})$ compatible bipartitions (as compared to $2^{|\mathcal {X}|-1}-1$ bipartitions). Intuitively, to enumerate the set of compatible bipartitions $\mathcal {B}(\mathcal {G})$, for each tree $G_i$, denoting $\textrm{ch}(\textrm{r}(G_i)) = \{v_\mathrm {\ell }, v_\textrm{r}\}$, we try all possibilities for placing $L(G_{v_\mathrm {\ell }})$ and $L(G_{v_\textrm{r}})$ in a bipartition $\{ {\mathcal {X}}_\ell ,{\mathcal {X}}_r \}$: either both in ${\mathcal {X}}_\ell$, or both in ${\mathcal {X}}_r$, or one in ${\mathcal {X}}_\ell$ and the other in ${\mathcal {X}}_r$. While each compatible bipartition corresponds to such a “split”, conversely, not all splits lead to a compatible bipartition, and those need to be filtered out. The case for $|\mathcal {G}| = 2$ is illustrated in Fig. 6.(1-2).

In the following sections, we will successively show how to compute solutions to the problem with some restrictions on the history, allowing us to solve the MinSynSupertree Problem.

We will consider the following two cases for the synteny content at $v = \varPsi _{H}(r(T))$: either $x(v) \subseteq x(\mathcal {G})$ or $x(v) \not \subseteq x(\mathcal {G})$. We define below the cost of a history corresponding to $\mathcal {G}$ where *b* determines which case we are considering.

#### Definition 7

**(Cost of History)** Let $\mathcal {G}= \{G_1, G_2, \dots , G_k\}$ be a tree set on ${\mathcal {X}}$ ($|{\mathcal {X}}| \ge 2$). Let $\{ {\mathcal {X}}_\ell ,{\mathcal {X}}_r \}$ be a compatible bipartition of ${\mathcal {X}}$, *b* be a boolean, and $\sigma \in \textrm{V}(S^*)$. We define $c(\mathcal {G},b,\sigma ,\{ {\mathcal {X}}_\ell ,{\mathcal {X}}_r \})$ (respec. $c(\mathcal {G},b,\sigma )$) as the minimum cost of any history $\mathcal {H}=\langle H, e, x, s\rangle$ explicating any synteny supertree *T* of $\mathcal {G}$ such that $\mathcal {H}$ and *T* satisfy conditions 1 to 4 (respec. conditions 1 to 3) below where $v = \varPsi _{H}(r(T))$. Note that $c(\mathcal {G},b,\sigma )$ is also defined in the same way for $|{\mathcal {X}}| = 1$.

1. $$s(v) = \sigma $$
2. $$ {\left\{ \begin{array}{ll} x(v) \subseteq x(\mathcal {G}) & \text { if}~b =~\textit{False}\\ x(v) \not \subseteq x(\mathcal {G}) & \text { otherwise} \end{array}\right. }$$
3. $$\left( (\cup _{\xi \in ({\mathcal {X}}^{input} - {\mathcal {X}})}x(\xi )) \cap x(\mathcal {G})\right) \subseteq x(v)$$
4. The bipartition of *r*(*T*) is $\{ {\mathcal {X}}_\ell ,{\mathcal {X}}_r \}$

By making sure that any gene family appearing both in the current restriction ($\varGamma \in x(\mathcal {G})$) and in any other part of the input ($\varGamma \in x(\xi )$ for $\xi \in {\mathcal {X}}^{input} - {\mathcal {X}}$) is included in the content of *v*, Condition 3 ensures that each gene family is gained exactly once in the history.

We are now ready to present our pseudocode, Algorithm 1, solving MinSynSupertree when called with input $(\mathcal {G}^{input}, S^*)$. The correctness of Algorithm 1 follows from the proofs of Theorems 2 to 6 that allow us to compute $c(\mathcal {G}, b, \sigma )$ on line 11 and from the fact that $\min _{T \in \mathbb {T}(\mathcal {G}^{input})} \{c^{\textrm{min}}(T, S)\} = \min _{\sigma \in V(S^*)}\{c(\mathcal {G}^{input}, \textit{False},\sigma )\}$ by definition.

**Algorithm 1:**
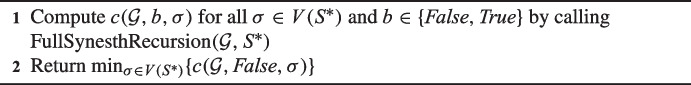
FullSynesth$$(\mathcal {G}, S^*)$$.

**Algorithm 2:**
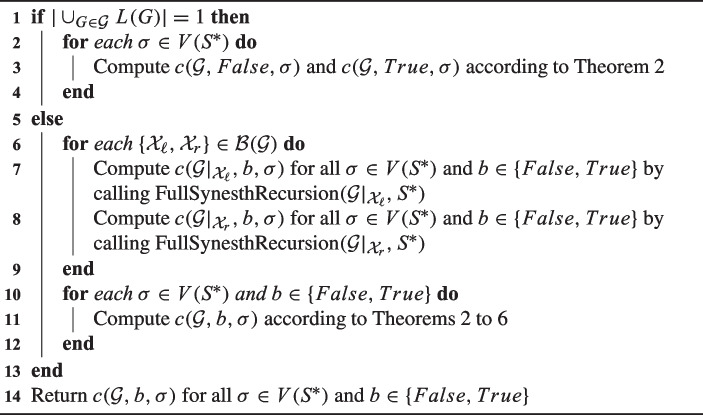
FullSynesthRecursion$$(\mathcal {G}, S^*)$$.

The next theorem states the main step of the recurrence algorithm.

#### Theorem 2

**(Testing bipartitions)** Let $\mathcal {G}= \{G_1, G_2, \dots , G_k\}$ be a tree set on ${\mathcal {X}}$. Let $\sigma \in S^*$ and *b* be a boolean. Then,

$$\begin{aligned}&\text {If } |{\mathcal {X}}| = 1,  &   c(\mathcal {G},b,\sigma ) = {\left\{ \begin{array}{ll} 0 & \text {if} \,\, \sigma = s(r(G_1)) \,\,\text {and}\,\, b \,\, \text {is False}\\ \infty &  \text {otherwise} \end{array}\right. }\\&\text {Otherwise, }  &   c(\mathcal {G},b,\sigma ) = \min _{\begin{array}{c} \{ {\mathcal {X}}_\ell ,{\mathcal {X}}_r \} \in {\mathcal {B}}(\mathcal {G}) \end{array}}\{c(\mathcal {G},b,\sigma ,\{ {\mathcal {X}}_\ell ,{\mathcal {X}}_r \})\} \end{aligned}$$

#### Proof

If $|{\mathcal {X}}| = 1$, then the optimal synteny supertree *T* of $\mathcal {G}$ is restricted to a single leaf $r(G_1)$, and the result is correct by definition of a history. In fact, if $\sigma \ne s(G_1)$ or *b* is true then there exists no history explicating *T* satisfying conditions 1 to 3 of Definition 7 as the item 2 of Definition 4 can’t be respected in that case.

Otherwise, let $\langle H, e, x, s\rangle$ be a history of cost $c(\mathcal {G},b,\sigma )$ explicating a synteny supertree *T* of $\mathcal {G}$ and satisfying conditions 1 to 3 of Definition 7 (notice that such a history always exists). Notice that as $|{\mathcal {X}}| \ge 2$, *r*(*T*) is a binary node. By definition, the bipartition $\{L(T_{v_\ell }), L(T_{v_r})\}$ of *r*(*T*) is in ${\mathcal {B}}(\mathcal {G})$. Therefore, the result is correct because, by Definition 7, $c(\mathcal {G},b,\sigma ,\{L(T_{v_\ell }), L(T_{v_r}))\} = c(\mathcal {G},b,\sigma )$. $\square$

### Syntenies

It is shown in [14] that, for computing the minimum cost of a history *H* explicating a given synteny supertree *T* for a set $\{G_1^{input}, G_2^{input}, \dots , G_k^{input}\}$ of consistent gene trees, it is sufficient to place the gain event for each gene family $\varGamma _i$ corresponding to $G_i^{input}$ so as to be the parent of the lowest common ancestor of the leaves they appear in, which corresponds to $\varPsi _H(r(G_i^{input}))$. From now on, we will assume that this is the case for all histories.

The next lemma, following from Lemma 2 in [14], states that only two synteny contents (i.e. one for each possible value of *b*) have to be tested at each internal node of the synteny supertree being constructed. The dummy gene $\varGamma ^*$ is use in the case $b={\textit{True}}$ (i.e. $x(v) \not \subseteq x(\mathcal {G})$), avoiding to test all possible subsets of $\mathcal {F}$. In fact, the lemma shows that choosing any possible subset satisfying Condition 3 of Definition 7 and such that $x(v) \not \subseteq x(\mathcal {G})$ leads to the same cost.

#### Lemma 3

**(Synteny contents)** Let $\mathcal {G}= \{G_1, G_2, \dots , G_k\}$ be a tree set on ${\mathcal {X}}$ ($|{\mathcal {X}}| \ge 2$). Let $\{ {\mathcal {X}}_\ell ,{\mathcal {X}}_r \}$ be a compatible bipartition of ${\mathcal {X}}$ and *b* be a boolean. Let $gain(\mathcal {G},{\mathcal {X}}') = \{\varGamma _i \mid (i \in \{1,\dots ,k\}) \wedge (r(G_i^{input}) \in V(G_i|_{{\mathcal {X}}'}))\}$ and

$$\begin{aligned} x^{syn}(\mathcal {G},b,{\mathcal {X}}_\ell ,{\mathcal {X}}_r)&={\left\{ \begin{array}{ll} x(\mathcal {G}) - gain(\mathcal {G},{\mathcal {X}}_\ell ) - gain(\mathcal {G},{\mathcal {X}}_r) \, \cup \, \{\varGamma ^*\} & \text {if { b} is True} \\ x(\mathcal {G}) - gain(\mathcal {G},{\mathcal {X}}_\ell ) - gain(\mathcal {G},{\mathcal {X}}_r) & \text {otherwise} \end{array}\right. } \end{aligned}$$

There exists a history $\langle H, e, x, s\rangle$ of cost $c(\mathcal {G},b,\sigma ,\{ {\mathcal {X}}_\ell ,{\mathcal {X}}_r \})$ explicating a synteny supertree *T* of $\mathcal {G}$ and satisfying conditions 1 to 4 of Definition 7 such that $x(\varPsi _H(r(T)))= x^{syn}(\mathcal {G},b,{\mathcal {X}}_\ell ,{\mathcal {X}}_r)$.

#### Proof

Notice first that a history explicating a synteny supertree *T* of $\mathcal {G}$ and satisfying conditions 1 to 4 of Definition 7 exists by definition given that the gene trees in $\mathcal {G}$ are consistent and that $\{ {\mathcal {X}}_\ell ,{\mathcal {X}}_r \}$ is compatible. Let $\mathcal {H}= \langle H, e, x, s\rangle$ be any such history. Note that $\varPsi _H(r(T))$ is a binary node as *r*(*T*) is binary (because $|{\mathcal {X}}| \ge 2$). Let $v_l$ and $v_r$ be the two children of $\varPsi _H(r(T))$. By definition, $gain(\mathcal {G},{\mathcal {X}}_\ell )$ (respect. $gain(\mathcal {G},{\mathcal {X}}_r)$) is the set of gene families that are gained in the subtree $H_{v_l}$ (respect. $H_{v_r}$) of $\mathcal {H}$.

As each gene family is gained only once in the history, $x(\varPsi _H(r(T)))$ must contain all gene families in $x(\mathcal {G})$ except those gained under $\varPsi _H(r(T))$. Thus,

2.1 $$\begin{aligned} x(\mathcal {G}) - gain(\mathcal {G},{\mathcal {X}}_\ell ) - gain(\mathcal {G},{\mathcal {X}}_r) \subseteq x(\varPsi _H(r(T)))\end{aligned}$$

2.2 $$\begin{aligned} (gain(\mathcal {G},{\mathcal {X}}_\ell ) \cup gain(\mathcal {G},{\mathcal {X}}_r)) \cap x(\varPsi _H(r(T))) = \emptyset \end{aligned}$$

- Case $b =$ False : In that case,
  2.3 $$\begin{aligned} x(\varPsi _H(r(T)))&\subseteq x(\mathcal {G})  &   \text {by definition~7}\end{aligned}$$
  2.4 $$\begin{aligned} x(\varPsi _H(r(T)))&\subseteq x(\mathcal {G}) - gain(\mathcal {G},{\mathcal {X}}_\ell ) - gain(\mathcal {G},{\mathcal {X}}_r)  &   \text {by (2.2)} \end{aligned}$$
  By (2.1) and (2.4), we conclude that
  $$\begin{aligned} x(\varPsi _H(r(T)))&= x(\mathcal {G}) - gain(\mathcal {G},{\mathcal {X}}_\ell ) - gain(\mathcal {G},{\mathcal {X}}_r)\\&=x^{syn}(\mathcal {G},b,{\mathcal {X}}_\ell ,{\mathcal {X}}_r) \end{aligned}$$
- Case $b =$ True : In that case, $x(\varPsi _H(r(T))) \not \subseteq x(\mathcal {G})$ by definition 7. Then there is a non empty synteny content $X \not \subseteq x(\mathcal {G})$, such that $x(\varPsi _H(r(T))) = x(\mathcal {G}) - gain(\mathcal {G},{\mathcal {X}}_\ell ) - gain(\mathcal {G},{\mathcal {X}}_r) \, \cup \, X$. Assume $\langle H, e, x, s\rangle$ is such a history of minimum cost $c(\mathcal {G},b,\sigma ,\{ {\mathcal {X}}_\ell ,{\mathcal {X}}_r \})$. By Lemma 2 in [14], for any non empty synteny content $X'$ such that $X' \ne X$ and $X'\not \subseteq x(\mathcal {G})$, there exists a history $\langle H', e', x', s'\rangle$ of minimum cost $c(\mathcal {G},b,\sigma ,\{ {\mathcal {X}}_\ell ,{\mathcal {X}}_r \})$ satisfying the same conditions such that $x(\varPsi _{H'}(r(T))) = x(\mathcal {G}) - gain(\mathcal {G},{\mathcal {X}}_\ell ) - gain(\mathcal {G},{\mathcal {X}}_r) \, \cup \, X'$. The result follows by choosing $X' = \{\varGamma ^*\}$.

In both cases, we have $x(\varPsi _H(r(T))) = x^{syn}(\mathcal {G},b,{\mathcal {X}}_\ell ,{\mathcal {X}}_r)$. $\square$

**Fig. 7:**
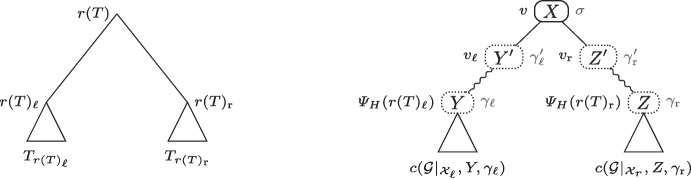
A decomposition for a history *H* (on the right) explicating a synteny tree *T* on the left. The notations illustrate those introduced in Definition 8. The wavy lines correspond to paths. This figure was adapted from [14, Figure 2]

We next introduce the notations allowing to decompose a history explicating a synteny tree, as illustrated in Fig. 7. Notice that, in Definition 8, the booleans *y* and *z* will be used later (Theorem 5) to define the syntenies *Y* and *Z*, respectively.

#### Definition 8

Let $\mathcal {G}= \{G_1, G_2, \dots , G_k\}$ be a tree set on ${\mathcal {X}}$ ($|{\mathcal {X}}| \ge 2$). Let $event \in \{\textrm{Spe},\textrm{Dup},\textrm{Cut},\textrm{TrDup},\textrm{TrCut}\}$, $\sigma ,\gamma _\mathrm {\ell }, \gamma _\mathrm {\ell }', \gamma _\textrm{r}, \gamma _\textrm{r}' \in \textrm{V}(S^*)$, $\{ {\mathcal {X}}_\ell ,{\mathcal {X}}_r \}$ be a compatible bipartition of ${\mathcal {X}}$, *y*, *z* be two booleans and $X,Y',Z' \subseteq \mathcal {F}$. We define $c(\mathcal {G},\sigma ,\{ {\mathcal {X}}_\ell ,{\mathcal {X}}_r \},event,X, Y', \gamma _\mathrm {\ell }',y, \gamma _\mathrm {\ell },Z',\gamma _\textrm{r}',z, \gamma _\textrm{r})$ (respec. $c(\mathcal {G},\sigma ,\{ {\mathcal {X}}_\ell ,{\mathcal {X}}_r \},event,X,y,z)$) as the minimum cost among the costs of any history $\mathcal {H}=\langle H, e, x, s\rangle$ explicating any synteny supertree *T* of $\mathcal {G}$ such that $\mathcal {H}$ and *T* satisfy conditions 1 to 9 (respec. 1 to 6) where $v = \varPsi _{H}(r(T))$ if such a history exists, and $\infty$ otherwise.

1. $$e(v) = event$$;
2. The bipartition of *r*(*T*) is $\{ {\mathcal {X}}_\ell ,{\mathcal {X}}_r \}$;
3. $$s(v) = \sigma $$, $$x(v) = X$$;
4. $${\left\{ \begin{array}{ll} x(\varPsi _{H}(r(T)_\ell )) \subseteq x(\mathcal {G}|_{{\mathcal {X}}_\ell }) & \text { if}~y =~False\\ x(\varPsi _{H}(r(T)_\ell )) \not \subseteq x(\mathcal {G}|_{{\mathcal {X}}_\ell }) & \text { otherwise;} \end{array}\right. }$$
5. $${\left\{ \begin{array}{ll} x(\varPsi _{H}(r(T)_r)) \subseteq x(\mathcal {G}|_{{\mathcal {X}}_r}) & \text { if}~z =~False\\ x(\varPsi _{H}(r(T)_r)) \not \subseteq x(\mathcal {G}|_{{\mathcal {X}}_r}) & \text { otherwise;} \end{array}\right. }$$
6. $$\left( (\cup _{\xi \in ({\mathcal {X}}^{input} - {\mathcal {X}})}x(\xi )) \cap x(\mathcal {G})\right) \subseteq x(v)$$;
7. $$s(\varPsi _H(r(T)_\ell )) = \gamma _\ell $$, $$s(\varPsi _H(r(T)_r)) = \gamma _r$$;
8. $$s(v_\ell ) = \gamma '_\ell $$, $$x(v_\ell ) = Y'$$;
9. $$s(v_r) = \gamma '_r$$, $$x(v_r) = Z'$$.

#### Theorem 3

**(Testing all possible syntenies)** Let $\mathcal {G}= \{G_1, G_2, \dots , G_k\}$ be a tree set on ${\mathcal {X}}$ ($|{\mathcal {X}}| \ge 2$). Let $\{ {\mathcal {X}}_\ell ,{\mathcal {X}}_r \}$ be a compatible bipartition of ${\mathcal {X}}$, $\sigma \in V(S^*)$, *b* be a boolean and let $X = x^{syn}(\mathcal {G},b,{\mathcal {X}}_\ell ,{\mathcal {X}}_r)$. Then,

$$\begin{aligned} c(\mathcal {G},b,\sigma ,\{ {\mathcal {X}}_\ell ,{\mathcal {X}}_r \}&)= \\  &\min _{\begin{array}{c} y\in \{False,True\}\\ z \in \{False,True\}\\ event \in \{\textrm{Spe},\textrm{Dup},\textrm{Cut},\textrm{TrDup},\textrm{TrCut}\} \end{array}}\nonumber \begin{aligned}\{c(\mathcal {G},\sigma ,\{ {\mathcal {X}}_\ell ,{\mathcal {X}}_r \},event,X,y,z)\} \end{aligned} \end{aligned}$$

#### Proof

Let $\mathcal {H}= \langle H, e, x, s\rangle$ be a history of minimum cost $c(\mathcal {G},b,\sigma ,\{ {\mathcal {X}}_\ell ,{\mathcal {X}}_r \})$ explicating a synteny supertree *T* of $\mathcal {G}$ and satisfying conditions 1 to 4 of Definition 7 such that $x(\varPsi _H(r(T))) = x^{syn}(\mathcal {G},b,{\mathcal {X}}_\ell ,{\mathcal {X}}_r)$. Recall that such a history exists by Lemma 3.

As $|{\mathcal {X}}| \ge 2$, $\varPsi _H(r(T))$ is a binary node and thus the possible events for $\varPsi _H(r(T))$ are in the set of binary events $\{\textrm{Spe},\textrm{Dup},\textrm{Cut},\textrm{TrDup},\textrm{TrCut}\}$. Let $y = \llbracket x(\varPsi _H(r(T)_\ell )) \not \subseteq x(\mathcal {G}|_{{\mathcal {X}}_{\ell }}) \rrbracket$ and $z = \llbracket x(\varPsi _H(r(T)_r)) \not \subseteq x(\mathcal {G}|_{{\mathcal {X}}_{r}}) \rrbracket$. The cost of $\mathcal {H}$ is $c(\mathcal {G},\sigma ,\{ {\mathcal {X}}_\ell ,{\mathcal {X}}_r \},e(\varPsi _H(r(T))),x^{syn}(\mathcal {G},b,{\mathcal {X}}_\ell ,{\mathcal {X}}_r),y,z)$ by Definition 8 and the result follows. $\square$

### Events

For each *event*, the cost $c(\mathcal {G},\sigma ,\{ {\mathcal {X}}_\ell ,{\mathcal {X}}_r \},event,X,y,z)$ is given in the next theorem, which follows from Definition 8 and Theorem 1 in [14]. Intuitively, referring to Fig. 7, it is obtained by considering all possibilities for $\gamma '_\ell$, $Y'$, $\gamma '_r$, $Z'$ given the event ( **Spe** , **Dup** , **Cut** , **TrDup** , or **TrCut** ), synteny content *X* and species label $\sigma$ at *v*. For example if $e(v) = \textrm{Spe}$, by Definition 1, $Y' = Z' = X$ and either $\gamma '_\ell = \sigma _\ell$ and $\gamma '_r = \sigma _r$, or $\gamma '_\ell = \sigma _r$ and $\gamma '_r = \sigma _\ell$.

#### Theorem 4

**(Testing all possible events)** Let $\mathcal {G}= \{G_1, G_2, \dots , G_k\}$ be a tree set on ${\mathcal {X}}$ ($|{\mathcal {X}}| \ge 2$). Let $B = \{ {\mathcal {X}}_\ell ,{\mathcal {X}}_r \}$ be a compatible bipartition of ${\mathcal {X}}$, $\sigma \in S^*$, *y*, *z* be booleans and $X \subseteq \mathcal {F}$. Then,

$$\begin{aligned}&{\textbf {Spe: }} c(\mathcal {G},\sigma ,\{ {\mathcal {X}}_\ell ,{\mathcal {X}}_r \},\textrm{Spe},X,y,z)=\\  &\hspace{13.4 mm} {\left\{ \begin{array}{ll} \infty & \text {if} \,\, \sigma \in L(S^*)\\ \min _{\begin{array}{c} \gamma _\mathrm {\ell }, \gamma _\textrm{r}\not < \sigma \\ \end{array}}\begin{aligned} \big \{ & c(\mathcal {G},\sigma ,B,\textrm{Spe},X, X, \sigma _\mathrm {\ell }, y, \gamma _\mathrm {\ell }, X, \sigma _\textrm{r}, z, \gamma _\textrm{r}), \\ & c(\mathcal {G},\sigma ,B,\textrm{Spe}, X, X, \sigma _\textrm{r}, y, \gamma _\mathrm {\ell }, X, \sigma _\mathrm {\ell }, z, \gamma _\textrm{r})\big \} \end{aligned} & \text {otherwise} \end{array}\right. } \end{aligned}$$

$$\begin{aligned}&{\textbf {Dup: }} c(\mathcal {G},\sigma ,\{ {\mathcal {X}}_\ell ,{\mathcal {X}}_r \},\textrm{Dup},X,y,z)=\\  &\hspace{31.5 mm}\min _{\begin{array}{c} \gamma _\mathrm {\ell }, \gamma _\textrm{r}\not < \sigma \end{array}} \begin{aligned} \big \{&c(\mathcal {G},\sigma ,B,\textrm{Dup}, X, X \cap x(\mathcal {G}|_{{\mathcal {X}}_\ell }), \sigma , y, \gamma _\mathrm {\ell }, X, \sigma , z, \gamma _\textrm{r}), \\&c(\mathcal {G},\sigma ,B,\textrm{Dup}, X, X, \sigma , y, \gamma _\mathrm {\ell }, X \cap x(\mathcal {G}|_{{\mathcal {X}}_r}), \sigma , z, \gamma _\textrm{r})\big \} \end{aligned} \end{aligned}$$

$$\begin{aligned}&{\textbf {Cut: }}c(\mathcal {G},\sigma ,\{ {\mathcal {X}}_\ell ,{\mathcal {X}}_r \},\textrm{Cut},X,y,z)=\\  &{\left\{ \begin{array}{ll} \infty \hspace{73 mm} \text {if} \,\,x(\mathcal {G}|_{{\mathcal {X}}_\ell }) \cap x(\mathcal {G}|_{{\mathcal {X}}_r}) \ne \emptyset \\ \min _{\begin{array}{c} \\ \gamma _\mathrm {\ell }, \gamma _\textrm{r}\not < \sigma \end{array}}\hspace{59.7 mm}\text {otherwise}\\ \begin{aligned} \hspace{5 mm}\big \{ & c(\mathcal {G},\sigma ,B,\textrm{Cut}, X, X \cap x(\mathcal {G}|_{{\mathcal {X}}_\ell }), \sigma , y, \gamma _\mathrm {\ell }, X - x(\mathcal {G}|_{{\mathcal {X}}_\ell }), \sigma , z, \gamma _\textrm{r}), \\ & c(\mathcal {G},\sigma ,B,\textrm{Cut}, X, X - x(\mathcal {G}|_{{\mathcal {X}}_r}), \sigma , y, \gamma _\mathrm {\ell }, X \cap x(\mathcal {G}|_{{\mathcal {X}}_r}), \sigma , z, \gamma _\textrm{r})\big \} \end{aligned} \end{array}\right. } \end{aligned}$$

$$\begin{aligned}&{\textbf {TrDup: }}c(\mathcal {G},\sigma ,\{ {\mathcal {X}}_\ell ,{\mathcal {X}}_r \},\textrm{TrDup},X,y,z)= \\  &{\left\{ \begin{array}{ll} \infty \hspace{92 mm}\text {if}\,\, \sigma = r(S^*) \\ \min _{\begin{array}{c} \gamma _i \mathrel {\Vert }\sigma ; \gamma _\textrm{t}\not< \gamma _i,\sigma \\ \gamma _\textrm{k}\not < \sigma \end{array}}\hspace{72.3 mm}\text {otherwise}\\ \begin{aligned} \hspace{15 mm}\big \{ & c(\mathcal {G},\sigma ,B,\textrm{TrDup},X, X \cap x(\mathcal {G}|_{{\mathcal {X}}_\ell }), \gamma _i, y, \gamma _\textrm{t}, X, \sigma , z, \gamma _\textrm{k}), \\ & c(\mathcal {G},\sigma ,B,\textrm{TrDup},X, X, \sigma , y, \gamma _\textrm{k}, X \cap x(\mathcal {G}|_{{\mathcal {X}}_r}), \gamma _i, z, \gamma _\textrm{t})\big \} \end{aligned} \end{array}\right. } \end{aligned}$$

$$\begin{aligned}&{\textbf {TrCut: }}c(\mathcal {G},\sigma ,\{ {\mathcal {X}}_\ell ,{\mathcal {X}}_r \},\textrm{TrCut},X,y,z)\})= \\  &{\left\{ \begin{array}{ll}\infty \hspace{52 mm}\text {if}\,\, \sigma = r(S^*) \text {or}\,\, x(\mathcal {G}|_{{\mathcal {X}}_\ell }) \cap x(\mathcal {G}|_{{\mathcal {X}}_r}) \ne \emptyset \\ \min _{\begin{array}{c} \gamma _i \mathrel {\Vert }\sigma ; \gamma _\textrm{t}\not< \gamma _i,\sigma \\ \gamma _\textrm{k}\not < \sigma \end{array}} \hspace{32.4 mm}\text {otherwise}\\ \begin{aligned} \hspace{1 mm}\big \{ & c(\mathcal {G},\sigma ,B,\textrm{TrCut}, X, X \cap x(\mathcal {G}|_{{\mathcal {X}}_\ell }), \gamma _i, y, \gamma _\textrm{t}, X - x(\mathcal {G}|_{{\mathcal {X}}_\ell }), \sigma , z, \gamma _\textrm{k}), \\ &  c(\mathcal {G},\sigma ,B,\textrm{TrCut}, X, X - x(\mathcal {G}|_{{\mathcal {X}}_r}), \gamma _i, y, \gamma _\textrm{t}, X \cap x(\mathcal {G}|_{{\mathcal {X}}_r}), \sigma , z, \gamma _\textrm{k}), \\ &  c(\mathcal {G},\sigma ,B,\textrm{TrCut}, X, X \cap x(\mathcal {G}|_{{\mathcal {X}}_\ell }), \sigma , y, \gamma _\textrm{k}, X - x(\mathcal {G}|_{{\mathcal {X}}_\ell }), \gamma _i, z, \gamma _\textrm{t}), \\ &  c(\mathcal {G},\sigma ,B,\textrm{TrCut}, X, X - x(\mathcal {G}|_{{\mathcal {X}}_r}), \sigma , y, \gamma _\textrm{k}, X \cap x(\mathcal {G}|_{{\mathcal {X}}_r}), \gamma _i, z, \gamma _\textrm{t})\big \} \end{aligned} \end{array}\right. } \end{aligned}$$

### History Decomposition

We next explain how the cost of a history verifying the conditions of Definition 8, i.e. decomposed into paths and sub-histories as shown in Fig. 7, is computed.

We first need some preliminary notations. A node $v$ of a history such that $x(v) = X$ and $s(v) = \sigma$ is denoted $[X, \sigma ]$. Moreover, define a path from a node *v* to *w* of a history as an acyclic history whose root is *v* and whose only visible leaf is *w*, and define $\textrm{cPath}([X, \sigma ], [Y, \gamma ])$ as the minimum cost of any path from $v=[X, \sigma ]$ to $w=[Y, \gamma ]$.

Let $\mathcal {G}= \{G_1, G_2, \dots , G_k\}$ be a tree set on ${\mathcal {X}}$, *b* be a boolean, ${\mathcal {X}}' \subseteq {\mathcal {X}}$ and $X \subseteq \mathcal {F}$. We define

$$\begin{aligned} syn(\mathcal {G},X,b,{\mathcal {X}}') = {\left\{ \begin{array}{ll} X & \text {if }b,\\ x(\mathcal {G}|_{{\mathcal {X}}'}) & \text {otherwise}. \end{array}\right. } \end{aligned}$$

Referring to Fig. 7, this function is used to compute the optimal synteny contents *Y* and *Z* as shown in the following theorem.

#### Theorem 5

**(Cost of history decomposition)** Let $\mathcal {G}= \{G_1, G_2, \dots , G_k\}$ be a tree set on ${\mathcal {X}}$ ($|{\mathcal {X}}| \ge 2$). Let $event \in \{\textrm{Spe},\textrm{Dup},\textrm{Cut},\textrm{TrDup},\textrm{TrCut}\}$, $\sigma ,\gamma _\mathrm {\ell }, \gamma _\mathrm {\ell }', \gamma _\textrm{r}, \gamma _\textrm{r}' \in \textrm{V}(S^*)$, $\{ {\mathcal {X}}_\ell ,{\mathcal {X}}_r \}$ be a compatible bipartition of ${\mathcal {X}}$, *y*, *z* be two booleans and $X,Y',Z' \subseteq \mathcal {F}$.

If there exists a history verifying conditions 1 to 9 of Definition 8, then:

$$\begin{aligned}&c(\mathcal {G},\sigma ,\{ {\mathcal {X}}_\ell ,{\mathcal {X}}_r \}, event, X, Y', \gamma _\mathrm {\ell }', y, \gamma _\mathrm {\ell }, Z', \gamma _\textrm{r}', z, \gamma _\textrm{r})= \delta _{event}\\&\hspace{56 mm}\begin{aligned} \&+ \ (\textrm{cPath}([Y', \gamma _\mathrm {\ell }'], [Y, \gamma _\mathrm {\ell }]) + c(\mathcal {G}|_{{\mathcal {X}}_\ell }, y, \gamma _\mathrm {\ell })) \\&+ \ (\textrm{cPath}([Z', \gamma _\textrm{r}'], [Z, \gamma _\textrm{r}]) + c(\mathcal {G}|_{{\mathcal {X}}_r}, z, \gamma _\textrm{r})) \end{aligned} \nonumber \\&\text {where}\,\,Y = syn(\mathcal {G},Y',y,{\mathcal {X}}_\ell )\,\, \text {and} \,\,Z = syn(\mathcal {G},Z',z,{\mathcal {X}}_r). \end{aligned}$$

#### Proof

Let $\mathcal {H}= \langle H, e, x, s\rangle$ be a history explicating a synteny supertree *T* of $\mathcal {G}$ of cost $c(\mathcal {G},\sigma ,\{ {\mathcal {X}}_\ell ,{\mathcal {X}}_r \},event,X, Y', \gamma _\mathrm {\ell }', y, \gamma _\mathrm {\ell }, Z', \gamma _\textrm{r}', z, \gamma _\textrm{r})$ satisfying conditions 1 to 9 of Definition 8 .

According to the structure of $\langle H, e, x, s\rangle$ (see Fig. 7), its cost is the sum of the following costs where $v = \varPsi _{H}(r(T))$:

$\bullet$: $C_1$ : The cost of the path from *r*(*H*) to *v* (not shown in Fig. 7);
$\bullet$: $C_2$ : The cost of the event at node *v*;
$\bullet$: $C_3$ : The cost of the path from $v_\ell$ to $\varPsi _H(r(T)_\ell )$;
$\bullet$: $C_4$ : The cost of the sub-history rooted at $\varPsi _H(r(T)_\ell )$;
$\bullet$: $C_5$ : The cost of the path from $v_r$ to $\varPsi _H(r(T)_r)$;
$\bullet$: $C_6$ : The cost of the sub-history rooted at $\varPsi _H(r(T)_r)$.

Thus, $$c(\mathcal {G},\sigma ,\{ {\mathcal {X}}_\ell ,{\mathcal {X}}_r \},event,X, Y', \gamma _\mathrm {\ell }', y, \gamma _\mathrm {\ell }, Z', \gamma _\textrm{r}', z, \gamma _\textrm{r}) = \varSigma _{i=1}^{6}C_i$$.

$\bullet$ For any such minimum history, the cost of the path from *r*(*H*) to $\varPsi _H(r(T))$ is zero as this path may always be restricted to gain events. Thus, $C_1 = 0$.

$$\bullet $$ As $$e(v) = event$$, $$C_2 = \delta _{event}$$.

$\bullet$ $C_3$ corresponds to the cost of a minimum path from $v_\ell = [Y',\gamma '_\ell ]$ to $\varPsi _H(r(T)_\ell ) = [Y,\gamma _\ell ]$ for a certain *Y*. We show below that $Y= syn(\mathcal {G},Y',y,{\mathcal {X}}_\ell )$ leads to a path of minimum cost.

Notice that in Theorem 6 (on page 24), which shows how to compute the minimum cost of a path, the only occurrences of *X* and *Y* in the formula for the cost a path from $[X,\sigma ]$ to $[Y,\gamma ]$ is to compute $\llbracket X \not \subseteq Y \rrbracket$. Furthermore, the cost of the path, for a given $\sigma$ and $\gamma$, can only be greater (or equal) if $\llbracket X \not \subseteq Y \rrbracket = 1$ rather than if $\llbracket X \not \subseteq Y \rrbracket = 0$.

- If $y = False$, then $Y \subseteq x(\mathcal {G}|_{{\mathcal {X}}_\ell })$. If $Y' \not \subseteq Y$ then there exists $\varGamma \in Y'$ such that $\varGamma \notin Y$. In that case, $\varGamma \notin x(\mathcal {G}|_{{\mathcal {X}}_\ell })$ as otherwise $\varGamma$ would be gained twice in the history (once in an ancestor of $v_\ell$ and once in a descendant of $\varPsi _H(r(T)_\ell )$) and thus $Y' \not \subseteq x(\mathcal {G}|_{{\mathcal {X}}_\ell })$. Conversely, if $Y' \subseteq Y$, then $Y' \subseteq x(\mathcal {G}|_{{\mathcal {X}}_\ell })$ as $Y \subseteq x(\mathcal {G}|_{{\mathcal {X}}_\ell })$. Therefore,
  $$\begin{aligned} \llbracket Y' \not \subseteq Y \rrbracket = \llbracket Y' \not \subseteq x(\mathcal {G}|_{{\mathcal {X}}_\ell }) \rrbracket \Rightarrow \\ cPath([Y',\gamma _\ell '],[Y,\gamma _\ell ]) = cPath([Y',\gamma _\ell '],[x(\mathcal {G}|_{{\mathcal {X}}_\ell }),\gamma _\ell ]) \end{aligned}$$
- If $y = True$, then $Y \not \subseteq x(\mathcal {G}|_{{\mathcal {X}}_\ell })$. In that case there is no need to lose a gene family from $v_\ell$ to $\varPsi _H(r(T)_\ell )$ in the history because *Y* is allowed to contain every gene family contained in $Y'$. Thus, we can assume $\llbracket Y' \not \subseteq Y \rrbracket = 0$ to obtain a minimum path (see explanation above). As $\llbracket Y' \not \subseteq Y' \rrbracket = 0$, $cPath([Y',\gamma _\ell '],[Y,\gamma _\ell ]) = cPath([Y',\gamma _\ell '],[Y',\gamma _\ell ])$.

In both cases, the cost of this path is $cPath([Y',\gamma _\ell '],[syn(\mathcal {G},Y',y,{\mathcal {X}}_\ell ),\gamma _\ell ])$. In a similar way, $C_5 = cPath([Z',\gamma _r'],[syn(\mathcal {G},Z',z,{\mathcal {X}}_r),\gamma _r])$.

$\bullet$ $C_4$ corresponds to the cost of a minimum history $\langle H_\ell , e_\ell , x_\ell , s_\ell \rangle$ explicating the synteny supertree $T_{r(T)_\ell }$ of the set $\mathcal {G}|_{{\mathcal {X}}_{\ell }}$ on ${\mathcal {X}}_\ell$ such that $x(\varPsi _{H_\ell }(r(T)_\ell )) = x(\varPsi _{H}(r(T)_\ell ))$ and $s(\varPsi _{H_\ell }(r(T)_\ell )) = s(\varPsi _{H}(r(T)_\ell ))$, while $C_6$ corresponds to the cost of a minimum history $\langle H_r, e_r, x_r, s_r\rangle$ explicating the synteny supertree $T_{r(T)_r}$ of the set $\mathcal {G}|_{{\mathcal {X}}_{r}}$ on ${\mathcal {X}}_r$ such that $x(\varPsi _{H_r}(r(T)_r)) = x(\varPsi _{H}(r(T)_r))$ and $s(\varPsi _{H_r}(r(T)_r)) = s(\varPsi _{H}(r(T)_r))$. To conclude the proof, we need to show that $C_4 = c(\mathcal {G}|_{{\mathcal {X}}_\ell }, y, \gamma _\mathrm {\ell })$ and $C_6 = c(\mathcal {G}|_{{\mathcal {X}}_r}, z, \gamma _\textrm{r})$. For this purpose, all we have to do is to show that the two histories verify the conditions one to three of Definition 7. As the proof is the same for the two histories, let us check this fact for $\langle H_\ell , e_\ell , x_\ell , s_\ell \rangle$. As $s(\varPsi _{H_\ell }(r(T)_\ell )) = \gamma _\ell$ and $\mathcal {H}$ respects conditions 4 of Definition 8, conditions one and two are verified. It remains to verify Condition 3. By definition of a history, no gene family may be gained twice in $\mathcal {H}$. Thus:

5.1 $$\begin{aligned}&\bigl ((\cup _{\xi \in {\mathcal {X}}- {\mathcal {X}}_\ell }x(\xi )) \cap x(\mathcal {G}|_{{\mathcal {X}}{_\ell }})\bigr ) \subseteq x(\varPsi _{H}(r(T)_\ell )) \nonumber \\ \Rightarrow&\bigl ((\cup _{\xi \in {\mathcal {X}}- {\mathcal {X}}_\ell }x(\xi )) \cap x(\mathcal {G}|_{{\mathcal {X}}{_\ell }})\bigr ) \subseteq x(\varPsi _{H_\ell }(r(T)_\ell )) \\&\text {as}\,\, x(\varPsi _{H_\ell }(r(T)_\ell )) = x(\varPsi _{H}(r(T)_\ell )) \nonumber \end{aligned}$$

By Condition 6 of Definition 8, we have

5.2 $$\begin{aligned} \left( (\cup _{\xi \in ({\mathcal {X}}^{input} - {\mathcal {X}})}x(\xi )) \cap x(\mathcal {G})\right) \subseteq x(v) \nonumber \\ \Rightarrow \left( (\cup _{\xi \in ({\mathcal {X}}^{input} - {\mathcal {X}})}x(\xi )) \cap x(\mathcal {G}|_{{\mathcal {X}}_\ell }) \right) \subseteq x(v)\,\,  &   \,\,\text {as} \,\, x(\mathcal {G}|_{{\mathcal {X}}_\ell })) \subseteq x(\mathcal {G}) \end{aligned}$$

From (5.2) and the fact that a gene family cannot be gained twice in the history $\mathcal {H}$, we have

5.3 $$\begin{aligned}&\left( (\cup _{\xi \in ({\mathcal {X}}^{input} - {\mathcal {X}})}x(\xi )) \cap x(\mathcal {G}|_{{\mathcal {X}}_\ell }) \right) \subseteq x(\varPsi _{H}(r(T)_\ell )) \nonumber \\ \Rightarrow&\left( (\cup _{\xi \in ({\mathcal {X}}^{input} - {\mathcal {X}})}x(\xi )) \cap x(\mathcal {G}|_{{\mathcal {X}}_\ell }) \right) \subseteq x(\varPsi _{H_\ell }(r(T)_\ell )) \\  &\text {as} \,\, x(\varPsi _{H_\ell }(r(T)_\ell )) = x(\varPsi _{H}(r(T)_\ell )) \nonumber \end{aligned}$$

As $${\mathcal {X}}_\ell \subseteq {\mathcal {X}}\subseteq {\mathcal {X}}^{input} $$,

5.4 $$\begin{aligned} {\mathcal {X}}^{input} - {\mathcal {X}}_\ell = ({\mathcal {X}}^{input} - {\mathcal {X}}) \cup ({\mathcal {X}}- {\mathcal {X}}_\ell ) \end{aligned}$$

From (5.1), (5.3) and (5.4) we obtain

$$\begin{aligned} \left( (\cup _{\xi \in ({\mathcal {X}}^{input} - {\mathcal {X}}_\ell )}x(\xi )) \cap x(\mathcal {G}|_{{\mathcal {X}}_\ell }) \right) \subseteq x(\varPsi _{H_\ell }(r(T)_\ell )) \end{aligned}$$

Thus $\langle H_\ell , e_\ell , x_\ell , s_\ell \rangle$ satisfies Condition 3 of Definition 7. $\square$

We finally show how the minimum cost of a path is computed. Theorem 6 below follows from Theorem 2 in [14] describing all Pareto-optimal paths which, by definition, are all the paths that can be of minimum cost for a given event cost vector.

#### Theorem 6

**(Minimum cost of paths)** Let $X$ and $Y$ be two syntenies and $\sigma$ and $\gamma$ be two species. If $\sigma \le \gamma$, let $\varOmega (\sigma ,\gamma )= |\{(u,v) \in P_{S^*}(\sigma ,\gamma ) \mid v \notin V(S)\}|$. If $\sigma < \gamma$, denote $\textrm{ch}(\sigma ) = \{\sigma _s, \sigma _a\}$ such that $\sigma _s$ is separated from $\gamma$ and $\sigma _a$ is an ancestor of $\gamma$. If $\gamma < \sigma$, then $\textrm{cPath}([X, \sigma ], [Y, \gamma ]) = \infty$. Otherwise,

6.1 $$\begin{aligned}&\textrm{cPath}([X, \sigma ], [ Y, \gamma ]) = \min \{ \nonumber \\&\quad \delta _\textrm{Loss}\cdot \varOmega (\sigma , \gamma ) + \delta _\textrm{Loss}\cdot \llbracket X \not \subseteq Y \rrbracket ,  &   \text {if } \sigma \le \gamma \end{aligned}$$

6.2 $$\begin{aligned}&\quad \delta _\textrm{Loss}\cdot \varOmega (\sigma , \gamma ) + \min \{\delta _\textrm{Dup}, \delta _\textrm{Cut}\} \cdot \llbracket X \not \subseteq Y \rrbracket ,  &   \text {if } \sigma \le \gamma \text { and }\nonumber \\  &    &\gamma \text { is unsampled} \end{aligned}$$

6.3 $$\begin{aligned}&\quad \delta _\textrm{TrDup}+ \delta _\textrm{Loss}\cdot \llbracket \sigma \text { is sampled\hspace{1.0pt}} \rrbracket ,  &   \text {if } \sigma \mathrel {\Vert }\gamma \end{aligned}$$

6.4 $$\begin{aligned}&\quad \delta _\textrm{TrCut}+ \delta _\textrm{Loss}\cdot \llbracket (X \not \subseteq Y ) \wedge (\sigma \text { is sampled\hspace{1.0pt}}) \rrbracket ,  &   \text {if } \sigma \mathrel {\Vert }\gamma \end{aligned}$$

6.5 $$\begin{aligned}&\quad \delta _\textrm{TrDup}+ \delta _\textrm{Loss}\cdot \llbracket \sigma _a \text { is sampled\hspace{1.0pt}} \rrbracket \nonumber \\&\hspace{3.73em}+ \delta _\textrm{Loss}\cdot \llbracket \sigma _s \text { is sampled\hspace{1.0pt}} \rrbracket ,  &   \text {if } \sigma \le \gamma \end{aligned}$$

6.6 $$\begin{aligned}&\quad \delta _\textrm{TrCut}+ \delta _\textrm{Loss}\cdot \llbracket (X \not \subseteq Y) \wedge (\sigma _s \text { is sampled\hspace{1.0pt}}) \rrbracket \nonumber \\&\hspace{3.55em} + \delta _\textrm{Loss}\cdot \llbracket (\sigma _a \text { is sampled\hspace{1.0pt}}) \wedge (\sigma _a \ne \gamma ) \rrbracket ,  &   \text {if } \sigma \le \gamma \end{aligned}$$

6.7 $$\begin{aligned}&\quad \delta _\textrm{TrCut}+ \min \{\delta _\textrm{Dup}, \delta _\textrm{Cut}\},  &   \text {if } \gamma = r(S^*)\end{aligned}$$

6.8 $$\begin{aligned}&\quad 2\delta _\textrm{TrDup}+ \delta _\textrm{Loss}\cdot \llbracket \sigma \text { sampled\hspace{1.0pt}} \rrbracket ,  &   \text {if } \gamma \ne r(S^*)\end{aligned}$$

6.9 $$\begin{aligned}&\quad \min \{\delta _\textrm{TrDup},\delta _\textrm{TrCut}\} \cdot \llbracket \sigma \ne \gamma \rrbracket + \delta _\textrm{TrCut}  &   \text {if } \gamma \ne r(S^*)\}\end{aligned}$$

#### Proof

We below make the link between each line above, and the paths of Theorem 2 in [14]. We will not redefine all the notations introduced in [14]. For full details on the sets of paths $P_\textrm{TrDup}$, $P_\textrm{TrCut}^{\textrm{Left}}$, etc., the reader is invited to refer to the previous paper. The Pareto-optimal paths are separated, in that paper, into three sets $P_0$, $P_1$ and $P_2$ respectively corresponding to paths with zero, one and two transfers ($\textrm{TrDup}$ or $\textrm{TrCut}$). For each path of the three sets, we compute the number of each events and then compute the corresponding cost given $\delta$. In this proof, we omit the gain events as they do not contribute to the cost.

**Fig. 8:**
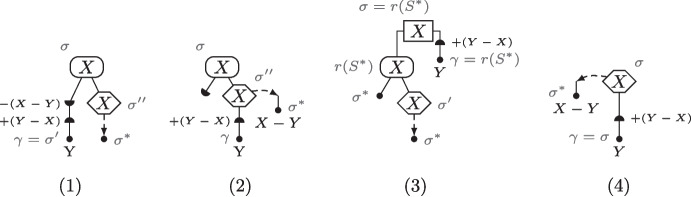
Some possible paths from $[X, \sigma ]$ to $[Y, \gamma ]$ detailed in the proof of Theorem 6. The notation is taken from [14]. The $\sigma ^*$ label denotes an unsampled species. The paths illustrated here are those missing from [14], due to an oversight in the original proof

- If $p \in P_0$, then $\sigma \le \gamma$. It follows that *c*(*p*) is given in line (6.1) if *p* contains a partial loss, and in line (6.2) if the path rather contains a duplication or a cut.
- For $p \in P_1$, *c*(*p*) is given in:
  - Line (6.3) if $\sigma \mathrel {\Vert }\gamma$ and $p \in P_\textrm{TrDup}$.
  - Line (6.4) if $\sigma \mathrel {\Vert }\gamma$ and $p \in P_\textrm{TrCut}^{\textrm{Left}} \cup P_\textrm{TrCut}^{\textrm{Right}}$.
  - Line (6.5) if $\sigma \le \gamma$ and $p \in P_\textrm{TrDup}$.
  - Line (6.6) if $\sigma \le \gamma$ and $p \in P_\textrm{TrCut}^{\textrm{Left}} \cup P_\textrm{TrCut}^{\textrm{Right}}$.
- The next three paths contain one transfer and were missing from [14] due to an oversight in the original proof. They are therefore detailed here.
  - Line (6.6) if $\sigma _a = \gamma$. The root of the path is a speciation node with children *v* and $v'$ such that $s(v) = \gamma$. There are two possible such paths. (1) $v'$ is a full $\textrm{TrCut}$ to any unsampled leaf separated from $s(v')$ and *v* is either the visible leaf $[Y,\gamma ]$ or a partial loss with the visible leaf $[Y,\gamma ]$ as child. See Fig. 8.(1). (2) $v'$ is a full loss and *v* is a $\textrm{TrCut}$ transferring the content $X-Y$ to any unsampled leaf separated from $\gamma$. See Fig. 8.(2).
  - Line (6.7) if $\gamma = r(S^*)$. The root of the path is a duplication or a cut event. One of its children is the visible leaf $[Y,\gamma ]$ and the other, *v*, is a speciation node. One of the children of *v* is an unsampled leaf (as $s(v) = r(S^*)$) and the other is a full $\textrm{TrCut}$ to the unsampled child of $r(S^*)$. See Fig. 8.(3).
  - Line (6.9) if $\sigma = \gamma \ne r(S^*)$. The root of the path is a $\textrm{TrCut}$ transferring the content $X-Y$ to any unsampled leaf separated from $\sigma$. The other child of the root is the visible leaf $[Y,\gamma ]$. See Fig. 8.(4).
- If $\gamma = r(S^*)$ then no transfer event can transfer to or from the species $\gamma$. This is reflected in Lines (6.8) and (6.9). In Theorem 2 in [14], other cases are forbidden for $P_2$, namely when $(\sigma \mathrel {\Vert }\gamma )$ and $X \subseteq Y$, or when $\sigma$ is unsampled. In fact, such cases never lead to Pareto-optimal histories. For brevity, we didn’t include those cases in our formulation, which doesn’t affect the minimality result. Note that the case $\sigma = r(S^*)$ was missing from [14] but such a path exists: the root is a speciation node with children *v* and $v'$ such that $v'$ is unsampled; *v* is a full $\textrm{TrCut}$ to the unsampled child of $r(S^*)$ and $v'$ is a transfer event (either $\textrm{TrCut}$ or $\textrm{TrDup}$) transferring the content $X \cap Y$ to $\gamma$. For $p \in P_2$, *c*(*p*) is given in:
  - Line (6.8) if $$p \in P^\textrm{TrDup}_\textrm{TrDup}$$.
  - Line (6.9) if $$p \in P^\textrm{TrCut}_\textrm{TrDup}\cup P^\textrm{TrCut}_\textrm{TrCut}$$.$$\square $$

### Time and Space Complexity

We can now state the time and space complexity of Algorithm 1.

#### Theorem 7

Let $k = |\mathcal {G}^{input}|$, $m=|\mathcal {X}^{input}|$ and $n= |V(S^*)|$. FullSynesth solves MinSynSupertree in $O((8m)^k \times (k^2 + n))$ time and $O((2m)^k \times (k+n))$ space.

#### Proof

We first show the time complexity. Note that line 2 of Algorithm 1 which consists in finding $\min _{\sigma \in V(S^*)}\{c(\mathcal {G}^{input}, False,\sigma )\}$ can be computed in time *O*(*n*) if $c(\mathcal {G}^{input}, False,\sigma )$ is known for each $\sigma \in V(S^*)$ (which it is at this step of the algorithm). We now show that line 1 of Algorithm 1 has a time complexity in $O((8m)^k \times (k^2 + n))$.

First, consider the number of different input set of trees $\mathcal {G}$ for which Algorithm 2 may be called, i.e. the number of possible restrictions of $\mathcal {G}^{input}$. Each tree *G* in $\mathcal {G}^{input}$ has at most *m* leaves and therefore at most $2m-1$ nodes. There are then at most $2m-1$ possible subtrees of *G* that may appear in $\mathcal {G}$ plus the possibility that no subtree of *G* appears in $\mathcal {G}$. There are thus at most $(2m)^k$ different restrictions of $\mathcal {G}^{input}$ and therefore Algorithm 2 will be called with at most $(2m)^k$ different inputs $\mathcal {G}$ by the algorithm. By storing the result of Algorithm 2 in cache for each possible input for which it is called, the result of any subsequent call with the same input can be retrieved in *O*(1) time.

The time complexity for a single call of Algorithm 2 with input $(\mathcal {G},S^*)$ (without considering the time complexity of the subsequent recursive calls) is:

- If $|\cup _{G \in \mathcal {G}}L(G)| = 1$, then Algorithm 2 first checks that $|\cup _{G \in \mathcal {G}}L(G)| = 1$ in *O*(*k*) and then computes $c(\mathcal {G}, b, \sigma )$ for each $\sigma \in V(S^*)$ and $b \in \{False,True\}$ according to Theorem 2 in *O*(*n*). In this case, the time complexity of the call is in $O(k + n)$.
- Otherwise, Algorithm 2 first checks that $|\cup _{G \in \mathcal {G}}L(G)| \ne 1$ in *O*(*k*). As shown by Lemma 2 in [19], the set of splits containing all bipartitions in ${\mathcal {B}}(\mathcal {G})$ can be computed in time $O(4^k)$ and $|{\mathcal {B}}(\mathcal {G})| \in O(4^k)$. After preprocessing the trees in $\mathcal {G}$, verifying if a given split forms a compatible bipartition can be done in time $O(k^2)$ by checking if there is a leaf in common between any tree in $\mathcal {G}|_{{\mathcal {X}}_\ell }$ and any tree in $\mathcal {G}|_{{\mathcal {X}}_r}$. Therefore, the set ${\mathcal {B}}(\mathcal {G})$ can be computed in $O(4^kk^2)$ time. For each bipartition in ${\mathcal {B}}(\mathcal {G})$, Algorithm 2 calls itself recursively (in *O*(1) time for each call as we consider here the time complexity of the current call without the subsequent calls) in lines 6 to 9. In lines 10 to 12, Algorithm 2 computes $c(\mathcal {G}, b, \sigma )$ according to Theorems 2 to 6 for each $\sigma \in V(S^*)$ and $b \in \{False,True\}$. For a given species $\sigma \in V(S^*)$ and $b \in \{False,True\}$, Theorem 2 tests all compatible bipartitions. For all compatible bipartitions, Theorem 3 tests a constant number of synteny contents, which are computed in time *O*(*k*). Note that the cost of a path can be computed in time *O*(*k*) according to Theorem 6, given that $S^*$ is preprocessed to allow $\varOmega (\sigma ,\gamma )$ and $\llbracket \sigma \mathrel {\Vert }\gamma \rrbracket$ to be computed in *O*(1) for all $\sigma ,\gamma \in V(S^*)$. For a given bipartition, species and synteny contents, Theorems 4 and 5 test all possible events and paths in time $O(kn^3)$ as $c(\mathcal {G}, False, \sigma )$ and $c(\mathcal {G},True, \sigma )$ are previously computed for each $\sigma \in V(S^*)$ and $b \in \{False,True\}$ in lines 6 to 9. This leads to a time complexity in $O(4^k \times (k^2 + kn^4))$ for a single call of Algorithm 2 with input $(\mathcal {G},S^*)$. However, it is possible to further optimize the algorithm to compute all minimal paths recursively, similarly to what is done in [21], to reduce the time complexity to $O(4^k \times (k^2 + n))$.

Therefore, each call of Algorithm 2 is computed in time $O(4^k \times (k^2 + n))$. As there are at most $(2m)^k$ different calls of Algorithm 2 during the execution of Algorithm 1, the time complexity of Algorithm 1 is $O((8m)^k \times (k^2 + n))$.

Regarding the space complexity, notice that the input $(\mathcal {G}^{input}, S^*)$ takes $O(km + n)$ space. The algorithm will then have to store at most $(2m)^k$ inputs and results, for all restrictions of $\mathcal {G}^{input}$. Each input $(\mathcal {G},S^*)$ can be stored in *O*(*k*) space by using pointers to nodes of the gene trees in $\mathcal {G}^{input}$ and a pointer to $S^*$. Each output takes *O*(*n*) space as we need to store $c(\mathcal {G}, b, \sigma )$ for every possible values of *b* and $\sigma$. Also, the space required to solve a given step of the recursion is in $O(k+n)$ because one may test each possible bipartition one after the other, saving only the optimal value for $c(\mathcal {G}, b, \sigma )$ for every *b* and $\sigma$. Therefore, FullSynesth solves MinSynSupertree using $O((2m)^k \times (k+n))$ space. $\square$

### Optimization from a Core Set of Trees

We show in this section how FullSynesth can be further optimized by considering, as shown in [19], a core set of trees rather than the whole set $\mathcal {G}^{input}$ of *k* gene trees. The idea is that the optimal bipartition found at a given step of the algorithm can be found from a subset of $\mathcal {G}^{input}$, as long as they span the set of leaves in the trees of $\mathcal {G}^{input}$. Given a set $\mathcal {G}$ of trees, a *core* of $\mathcal {G}$ is a subset $\mathcal {G}'$ of $\mathcal {G}$ such that$\cup _{G' \in \mathcal {G}'}L(G') = \cup _{G \in \mathcal {G}}L(G)$.

To solve MinSynSupertree, we may run FullSynesth on a core set of trees $\mathcal {G}'$ of $\mathcal {G}^{input}$ with the modification that when considering a bipartition $\{ {\mathcal {X}}_\ell ,{\mathcal {X}}_r \}$ compatible with a restriction of $\mathcal {G}'$ at a given step, we must make sure that $\{ {\mathcal {X}}_\ell ,{\mathcal {X}}_r \}$ is also compatible with the trees in $\mathcal {G}^{input} - \mathcal {G}'$. To do so, we may verify that $\{ {\mathcal {X}}_\ell ,{\mathcal {X}}_r \}$ is compatible with each tree $G \in \mathcal {G}^{input} - \mathcal {G}'$ by checking if $lca_G({\mathcal {X}}_\ell \cap L(G))$ and $lca_G({\mathcal {X}}_r \cap L(G) )$ are separated. This can be done in $O(k^2)$ time using the procedure described below after preprocessing the trees in $\mathcal {G}^{input}$ in a way allowing, for any two trees $G_1$ and $G_2$ in $\mathcal {G}^{input}$ and a node *v* of $G_1$, $lca_{G_2}(L({G_1}_v)\cap L(G_2))$ to be computed in *O*(1) time.

The pseudocode below uses an initial dummy node, called *dummyNode*, which is considered separated from any other node in any tree (including itself) and is such that $lca_{G}(\{dummyNode\} \cup V') = lca_{G}(V')$ for any tree *G*.

**Algorithm 3:**
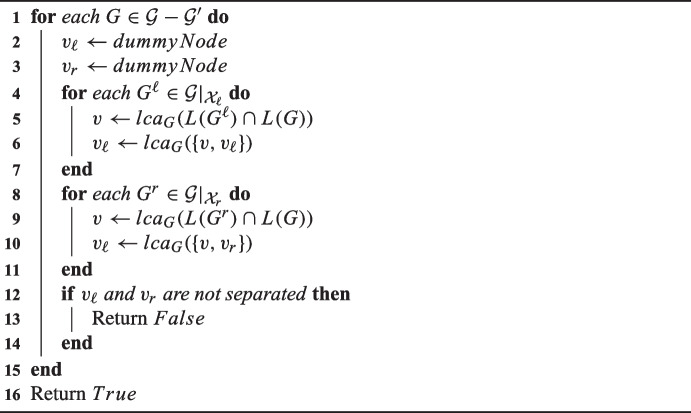
CompatibleBipartition$$(\mathcal {G}|_{{\mathcal {X}}_\ell }, \mathcal {G}|_{{\mathcal {X}}_r},\mathcal {G}^{input} - \mathcal {G}')$$.

The following result directly follows from Theorem 7 and the optimization described above.

#### Corollary 1

Let $k'$ be the size of a core set of $\mathcal {G}^{input}$, $m=|\mathcal {X}^{input}|$ and $n= |V(S^*)|$. FullSynesth solves MinSynSupertree in $O((8m)^{k'} \times (k^2 + n))$ time and $O((2m)^{k'} \times (k+n))$ space.

Note that the problem of finding a core set of minimum size is equivalent to the Minimum set cover problem and is thus an NP-hard problem. A simple heuristic to find a core set of a set $\mathcal {G}^{input}$ on $\mathcal {X}$ is to first choose the tree *G* in $\mathcal {G}^{input}$ with the most leaves to be in the core and then add at most $|\mathcal {X}| - |L(G)|$ trees from $\mathcal {G}^{input}$ to the core set, each containing at least one leaf not belonging to *G*.

## Results

**Fig. 9:**
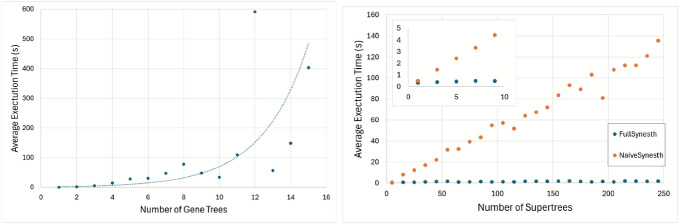
(Left): Average running time of FullSynesth on simulated datasets grouped according to the number of gene trees, keeping only samples containing more than 10 simulations. (Right): Average running time of FullSynesth and NaiveSynesth on simulated datasets grouped according to the number of possible supertrees. Each dot represents the average for a number of supertrees in an interval of size 10 around the dot. The scatter plot on top is a zoom on the first interval of the main scatter plot

We wanted to test the running time of FullSynesth on relevant datasets, obtained from evolutionary histories involving segmental events on syntenies. For this purpose, we developed *Syntesim*, a simulator generating evolutionary histories of species, gene families and syntenies. Starting from a synteny with a single gene belonging to an ancestral species, the simulation chooses, at each step, an event among Speciation, Extinction, Duplication, Cut, Transfer, Gain and Loss according to given rates on operations. In agreement with a classical assumption that transfers are less frequent than duplications [13], the rate of duplications has been chosen to be twice the rate of transfers. Moreover, we chose a lower rate for losses and cuts. In fact, the more losses and cuts, the more separated the gene trees are in their leafsets, and the more supertrees we obtain. As we show in Fig. 9, the number of supertrees obtained is already very large, preventing NaiveSynesth from terminating in a reasonable time.

The output of one simulation is a full history including a species tree *S*, a set $\mathcal {F}$ of gene families, a set $\mathcal {X}$ of syntenies, a set $\mathcal {G}$ of consistent gene trees for the gene families in $\mathcal {F}$, and a synteny tree *T* for $\mathcal {X}$ which is a supertree of the gene trees of $\mathcal {G}$. We used our simulator with the stopping criterion being to reach $|\varSigma | = 10$ species. We retained 2300 histories with $20 \le |\mathcal {X}| \le 30$.

We then ran FullSynesth on our dataset with the optimization from a set of core trees, as described in Section 6.6, on a desktop computer equipped with an AMD Ryzen 9 5900x processor and 64GB of RAM. The scatter plot to the left of Fig. 9 shows the results obtained by averaging the running time on inputs containing the same number of gene trees (size of $\mathcal {G}$), and keeping only the dots corresponding to at least 10 inputs. The figure reflects the increase in running time of FullSynesth, from 0.3 s for a single gene tree to about 10 min for 15 gene trees.

We then compared FullSynesth with *NaiveSynesth* which consists in solving MinSynSupertree by applying the original Synesth algorithm to each supertree for $\mathcal {G}$. As Synesth has a time complexity in $O(m (k+n))$^1^ [14] (where $k = |\mathcal {G}|$, $m=|\mathcal {X}|$ and $n= |V(S^*)|$) and as in worst case the number of synteny supertrees is equal to the number of rooted trees on *m* leaves, which equals $(2m-3)!!$, this way of solving MinSynSupertree has a time complexity in $O(m (k+n) (2m-3)!!)$.

In other words, the running time of NaiveSynesth is directly proportional to the number of supertrees. As the time complexity of FullSynesth does not directly depend on the number of supertrees, NaiveSynesth is clearly slower than FullSynesth if $|\mathbb {T}(\mathcal {G})|$ is large enough (in average NaiveSynesth takes more than 10 min to run if $|\mathbb {T}(\mathcal {G})|> 2000$). We thus restricted the comparison to low values of $|\mathbb {T}(\mathcal {G})|$. To this end, we implemented AllTrees [17] allowing to output the full set of binary supertrees for a given set of consistent trees. We then grouped our 2300 inputs according to the number of supertrees obtained from the set $\mathcal {G}$ of gene trees for each input.

While the number of supertrees does not directly appear in the worst-time complexity of FullSynesth, its running time may be affected by it. In fact, the more supertrees for $\mathcal {G}$, the more compatible bipartitions should be tested. For the dataset we considered, the running time of FullSynesth goes from an average of 0.3 s for less than 10 supertrees to an average of 2 min for more than 500 supertrees. However, as we can see from the right of Fig. 9, FullSynesth is always much faster on average than NaiveSynesth, even for fewer than 10 supertrees, and it is about 75 times faster on average for 250 supertrees.

## Conclusion

FullSynesth is a comprehensive tool for predicting the evolution of a set of homologous genomic regions through segmental events (duplications, gains, losses, cuts, horizontal gene transfers). It is a generalization of the gene/species tree reconciliation method taking a set of gene trees, rather than a single gene tree as input, and accounting for segmental, rather than individual, events. FullSynesth adds a key piece to the Synesth architecture since the synteny tree does not need to be known in advance. Synesth can however be part of a pipeline, called NaiveSynesth, testing each supertree in turn. We showed that FullSynesth is several orders of magnitude faster NaiveSynesth. As the two algorithms are exact, comparing their output is not required as they both return the same predictions.

From a complexity point of view, for a fixed number of gene trees, FullSynesth runs in polynomial time, while NaiveSynesth has a double factorial time complexity on the number of syntenies. However, there is a trade-off between time and space complexity as NaiveSynesth does not need to keep all supertrees in memory and can rather process each supertree in turn, which makes it more efficient in space (although for a fixed number of gene trees, both algorithms have a polynomial space complexity).

There are a number of possible future directions for improvements, probably the most important being to deal with the case of a set $\mathcal {G}= \{G_1, G_2, \dots , G_k\}$ of gene trees that are inconsistent, and thus not included in a supertree. For a given tree distance, one can be interested in finding a median tree, i.e. a tree on the leafset of $\mathcal {G}$ minimizing the sum of distances with the gene trees. This problem has been shown NP-hard for the D and DL reconciliation distance [22–24], and heuristics have been developed [25, 26]. Another way would be to find a consensus of gene trees in form of a sufficiently constrained phylogenetic network, such as a tree-child network, and design a strategy for building and reconciling a synteny tree from this network [27].

## Data Availability

No datasets were generated or analysed during the current study.

## Footnotes

1 Note that in [14] the factor *k* is omitted in the complexity because the number of gene families is considered constant.
